# Deciphering genetic causality between inflammatory bowel disease and periodontitis through bi-directional two-sample Mendelian randomization

**DOI:** 10.1038/s41598-023-45527-z

**Published:** 2023-10-30

**Authors:** Feiyan Yu, Yang Yang, Dongchao Wu, Minjing Chang, Chong Han, Qianqian Wang, Yi Li, Dongning He

**Affiliations:** 1 Shanxi Medical University School and Hospital of Stomatology, Taiyuan, China; 2 Shanxi Province Key Laboratory of Oral Diseases Prevention and New Materials, Taiyuan, China; 3 Shanxi Key Laboratory of Big Data for Clinical Decision, Shanxi Medical University, Taiyuan, China

**Keywords:** Periodontitis, Genetic linkage study

## Abstract

Inflammatory bowel disease (IBD) and periodontitis are reported to be closely associated; however, whether there is a causal association between them remains unclear. To explore the existence of this causality, this study applied a bidirectional two-sample Mendelian randomization (MR). The genetic variants were obtained from the summary statistics of genome-wide association studies of IBD, including its subtypes CD and UC, and periodontitis. 175, 148, 113, and six single-nucleotide polymorphisms were selected as instrumental variables for IBD, CD, UC, and periodontitis, respectively. In MR analysis, random-effects inverse-variance weighted was used as the primary method, and weighted median and MR Egger regression were applied as the complementary method. A series of sensitivity analyses were also conducted to ensure the reliability of the results. None of these analyses found a significant effect of genetically proxied IBD and its subtypes on periodontitis, and vice versa. Subsequent sensitivity analyses did not detect any horizontal pleiotropy and heterogeneity. Caution should be exerted when it comes to clinical relevance and further studies are needed to clarify the relationship between IBD and periodontitis.

## Introduction

Inflammatory bowel disease (IBD), mainly comprising Crohn’s disease (CD) and ulcerative colitis (UC), is a chronic, relapsing, and non-specific inflammatory disorder of the intestine^1^. CD usually affects the entire gastrointestinal tract and leads to transmural inflammation, whereas UC mainly affects the colon and rectum, causing inflammation restricted to the mucosal layer^2^. The suggested main cause of IBD lies in the interaction between potential pathogenic bacteria and the immune system of the host, which can be influenced by genetic and environmental factors^3^. In addition to intestinal inflammation, IBD patients also suffer extraintestinal manifestations among which periodontitis is a frequently reported oral manifestation^4^.

Periodontitis is a chronic inflammatory disease of periodontium, a collective term for the tissues that surround and support the dentition (gingiva, periodontal ligament, and alveolar bone), with an infectious origin^5^. The features of periodontitis include clinical attachment loss, alveolar bone absorption, periodontal pocketing, gingiva bleeding, and finally tooth loss, compromising mastication, esthetics, and life quality^6^. The pathogenesis of periodontitis has not been completely understood yet; however, a complex combination of genetic and environmental factors and dysregulated host immune response to oral microbes is widely accepted as the cause^7^. Furthermore, host genetic variants can affect the immune response to pathogens, increase susceptibility to the colonization of specific bacteria, and affect the threshold of dysbiosis as opposed to self-resolution in response to an acute change^8^.

Several lines of evidence have suggested the relevance between IBD and periodontitis. A higher prevalence of periodontitis was observed in patients with IBD than in controls in a case–control study (37.5% vs. 19.2%)^9^. Furthermore, periodontitis in patients with IBD presented more severe symptoms than in controls^10^. A previous meta-analysis, including eight case–control studies and one longitudinal cohort study, showed clear evidence for a higher prevalence of periodontitis in patients with IBD^11^. However, no evidence attributes this association to causality. From a medical and therapeutic standpoint, causality has more clinical value than mere association. As an example, if a causal effect of IBD on periodontitis does exist, the treatment of IBD may thus contribute to the improvement of periodontitis in IBD patients. Due to reverse causality, measurement error, and underlying bias, observational studies have limitations in causality inference^12^. Despite being adjusted, confounders, such as age, sex, smoking, and medications, may still interfere with meta-analyses and lead to biases. Randomized controlled trials (RCTs) are considered the gold standard for causality inference; however, RCTs on the causality between IBD and periodontitis in humans have so far not been conducted due to practicality, cost, and ethical considerations. Sufficient evidence to help understand whether IBD can cause periodontitis is still lacking.

Mendelian randomization (MR) provides a powerful strategy for causality inference. It achieves a randomization design similar to the one in RCTs through randomization inherent in gamete formation and therefore is not subject to confounding or reverse-causality bias^13^. In MR analysis, if genetic variants, in this case, single nucleotide polymorphisms (SNPs), associated with exposure link to a greater risk of outcome, a causal effect of the genetically proxied exposure on the outcome can be established. Taking advantage of published large-scale genome-wide association studies with no sample overlap, a two-sample MR analysis has great potential in causality inference with high statistical power^14^.

In this study, we used a two-sample MR framework to detect the potential causal association between IBD and periodontitis. The results can offer robust evidence in exploring the nature of the association between these two conditions.

## Materials and methods

### Study design

**Figure 1:**
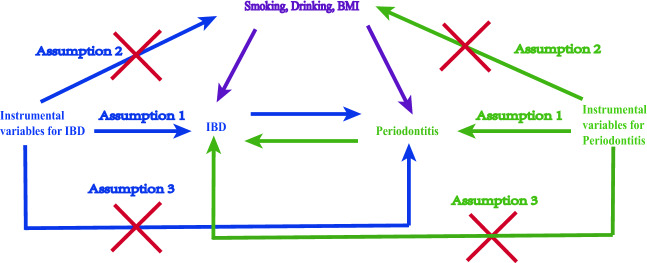
Schematic diagram of Mendelian randomization study. Instrumental variable assumption (1) The IVs robustly associate with exposure (relevance). (2) The IVs do not associate with confounders (exchangeability). (3) The IVs affect the outcome only through exposure rather than any other ways (exclusion restriction). *IBD:* inflammatory bowel disease, *BMI:* body mass index.

We applied two-sample MR analysis using instrumental variable (IV) estimation. IVs must meet the following three assumptions (Fig. 1): (1) IVs robustly associate with exposure (relevance). (2) IVs do not associate with confounders (exchangeability). (3) IVs affect the outcome only through exposure rather than any other ways (exclusion restriction)^15^. This study was approved by the human research ethics committee in the Shanxi Medical University School and Hospital of Stomatology (2022SLL029). This study adhered to the rigorous Strengthening the Reporting of Observational Studies in Epidemiology (STROBE) guideline for MR papers^16^.

### Data source

All datasets in this study are publicly available and show no sample overlap. Summary statistics of periodontitis were obtained from a genome-wide association study (GWAS) of European studies of the Gene-Lifestyle Interactions in Dental Endpoints (GLIDE) consortium, including 17,353 clinical periodontitis cases and 28,210 control cases^17^. Periodontitis cases were defined by the Centers for Disease Control and Prevention/American Academy of Periodontology or the Community Periodontal Index case definition or via study participant reports of the diagnosis of periodontitis^17^. GWAS for the periodontitis trait applied a mixed logistic model and adjusted for age, sex, and genetic principal components. Genotyping was performed on commercially available arrays (including the Affymetrix Genome-Wide Human SNP array, Illumina Human610 Quadv1_B, and Affymetrix UK BiLEVE Axiom array) and standard quality control checks were performed before imputation in this GWAS meta-analysis. Summary statistics for IBD (25,042 cases; 34,915 controls), UC (12,366 cases; 33,609 controls), and CD (12,194 cases; 28,072 controls) were derived from a GWAS of European participants^18^. The diagnosis of IBD was done using accepted endoscopic, histopathological, and radiological criteria. The study performed a fixed-effects meta-analysis; IBD and control cases in this GWAS were genotyped on the Human Core Exome v12.1. The details of the quality control method are provided in the original manuscript. Both data sources are briefly described in Table S1. The included GWASs obtained ethical review board approval and informed consent as described in the respective original manuscript.

### Instrument variants selection

We performed a set of quality control techniques to fulfill the first MR assumption. SNPs, acting as IVs in the present MR analysis, were selected at genome-wide significance (*P* < 5 × 10^−8^ for IBD and *P* < 5 × 10^−6^ for periodontitis), and palindromic variants were removed. The selected SNP unavailable in the outcome datasets was also removed. Then, we performed linkage disequilibrium (LD) clumping to ensure that SNPs were independent (clumping r^2^ cut-off = 0.001 and clumping window = 10,000 kb). SNP with an r^2^ larger than the stated threshold was retained. The strength of IVs was further examined using the F-statistic. SNPs with F-statistics < 10 were removed to avoid weak instrument bias for further verifying the relevance assumption. We also ruled out potential pleiotropy by manually searching all IVs in the PhenoScanner for detecting previously reported confounders, including smoking, drinking, and body mass index. We excluded IVs associated with these confounders from the following analysis.

### MR analysis

We harmonized summary statistics results to ensure that the effect allele of each SNP was consistent between exposure and outcome and to prohibit strand mismatch. This two-sample MR applied the random-effects inverse-variance weighted (IVW)^19^, the weighted median^20^ and the MR Egger regression^21^ to evaluate the causal effects of exposures on outcomes. With great statistical power and sensitivity to directional horizontal pleiotropy, the IVW method was treated as a fundamental analysis method. The result of the IVW method is robust if all IVs are valid or if any horizontal pleiotropy is balanced^22^.

### Sensitivity analysis

The violations of the second and third assumptions can occur through horizontal pleiotropy (the genetic variant affects the exposure and outcome but via different pathways). Sensitivity analysis can be used to uncover the possible violations of these assumptions. The Egger regression intercept was used to assess the presence of horizontal pleiotropy^21^. Intercept centered at the origin with a 95% confidence interval including the null reveals the absence of pleiotropy. The MR pleiotropy residual sum and outlier test were also used as complementary analyses to indicate the presence of pleiotropy^23^. We used the IVW method and MR Egger regression to detect heterogeneity, which was qualified through Cochrane's Q statistic (*P* < 0.1 indicates heterogeneity). A funnel plot was used for the visualization of heterogeneity in the causal estimates from different genetic instruments. The “leave-one-out” test was used to identify whether a single SNP strongly affected the causal estimate. Additionally, we also applied Latent Heritable Confounder Mendelian randomization (LHC-MR) as a sensitivity analysis^24^. This method appropriately uses genome-wide genetic markers to estimate bidirectional causal effects, direct heritability, confounder effect and population stratification while accounting for sample overlap. LHC-MR extends the standard two-sample MR by modeling a latent heritable confounder that affects both the exposure and outcome traits. It can differentiate SNPs based on their co-association to a pair of traits. Thus, the unbiased bidirectional causal effect between these two traits is estimated simultaneously as well as the confounder effect on each trait. All MR analyses were performed using R (version 4.0.3). *P* < 0.05 was considered statistically significant.

### Ethics approval

All methods were carried out following relevant guidelines and regulations. This study design was approved by the human research ethics committee in the Shanxi Medical University School and Hospital of Stomatology (2022SLL029).

### Consent to participate

The present study is based on summary-level data that have been made publicly available. In all original studies, participate consent had been obtained.

## Results

### Causal effects of IBD and its subtypes on periodontitis

#### Character of selected SNPs

The first MR assumption was fulfilled through the quality control techniques. We identified 187, 162, and 125 independent SNPs associated with IBD, CD, and UC with *P* < 5 × 10^−6^, respectively. No palindromic genetic variant was seen among these SNPs. Among them, 179 SNPs for IBD, 157 SNPs for CD, and 119 SNPs for UC were available in the summary statistic of periodontitis. All SNPs were independent in LD clumping. The F-statistic values for all these SNPs were all larger than 10, suggesting that the weak instrument bias cannot substantially affect the causal estimates. The search in the PhenoScanner database further showed that 4 IBD SNPs, 9 CD SNPs, and 6 UC SNPs were associated with previously reported confounders, and they were thus excluded from subsequent analyses. Finally, 175 SNPs for IBD, 148 SNPs for CD, and 113 SNPs for UC were incorporated into the MR and sensitive analyses. Detailed information about these SNPs is shown in Tables S2–S4.

#### MR and sensitivity analysis

**Figure 2:**
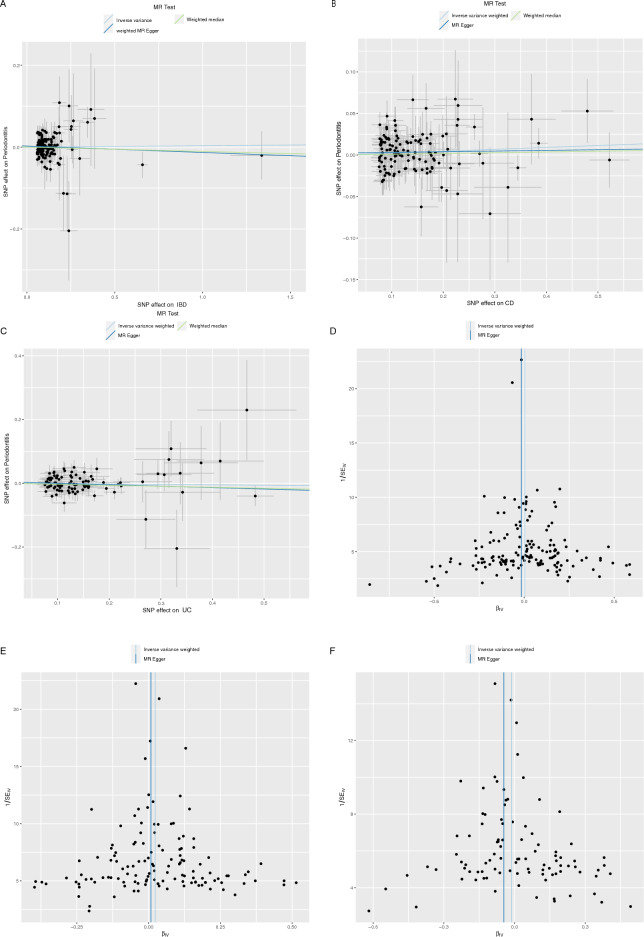
Scatter plot and Funnel plot of the causality of IBD, CD, and UC on periodontitis. (**A**) Scatter plot of the causal effect of IBD on periodontitis; (**B**) Scatter plot of the causal effect of CD on periodontitis; (**C**) Scatter plot of the causal effect of UC on periodontitis; (**D**) Funnel plot of the causal effect of IBD on periodontitis; (**E**) Funnel plot of the causal effect of CD on periodontitis; (**F**) Funnel plot of the causal effect of UC on periodontitis.

In the IVW analysis, the fundamental analysis, no statistically significant causal effects of genetically predicted IBD as a whole and its subtypes, CD and UC, on periodontitis were noted [IBD: odds ratio (OR) = 1.003, 95% CI = 0.977–1.030, *P* = 0.808; CD: OR = 1.023, 95% CI = 1.000–1.047, *P* = 0.051; UC: OR = 0.988, 95% CI = 0.958–1.019, *P* = 0.448] (Table 1, Fig. 2). The applied complementary MR approaches, the weighted median and MR Egger regression method, confirmed the results with point estimates slightly above and below 1 for the hypotheses—IBD affecting periodontitis—with highly overlapping CIs (Table 1, Fig. 2). The violations of the second and third assumptions were not found after statistical analysis. No heterogeneity was noted between the individual SNP of IBD and its subtypes in the heterogeneity analysis (IBD: *P* value of IVW method: 0.863, *P* value of MR Egger method: 0.858; CD: *P* value of IVW method: 0.514, *P* value of MR Egger method: 0.498; UC: *P* value of IVW method: 0.415, *P* value of MR Egger method: 0.405) (Table 2). No horizontal pleiotropy was detected in the Egger intercept test (IBD: intercept = 0.003, *P* = 0.439; CD: intercept = 0.002, *P* = 0.553; UC: intercept = 0.005, *P* = 0.428) (Table 3). The MR pleiotropy residual sum and outlier test outcomes showed that there were no horizontal pleiotropic outliers to distort the causality estimate. The “leave-one-out” test showed no outliers among these included SNPs. All sensitive analyses supported that there is no causal effect of genetically predicted IBD, UC, and CD on periodontitis. Figure 2 and Figs. S1–S3 show the funnel plots and leave-one-out analysis plots. LHC-MR also demonstrated no causal effect of IBD as a whole and its subtypes on periodontitis (IBD-Periodontitis: SE = 0.039, *P* = 0.858; UC-Periodontitis: SE = 0.071, *P* = 0.393; CD-Periodontitis: SE = 0.033, *P* = 0.470).

**Table 1:** MR estimates of assessing the causal association between IBD and periodontitis.

Outcome	Exposure	Method	OR	95% CI		*P-*value
Periodontitis	IBD	Inverse variance weighted	1.003	0.977	1.030	0.808
Periodontitis	IBD	MR Egger	0.984	0.932	1.040	0.576
Periodontitis	IBD	Weighted median	0.989	0.944	1.037	0.660
Periodontitis	CD	Inverse variance weighted	1.023	1.000	1.047	0.051
Periodontitis	CD	MR Egger	1.008	0.956	1.064	0.766
Periodontitis	CD	Weighted median	1.009	0.973	1.047	0.628
Periodontitis	UC	Inverse variance weighted	0.988	0.958	1.019	0.448
Periodontitis	UC	MR Egger	0.956	0.876	1.043	0.313
Periodontitis	UC	Weighted median	0.969	0.926	1.014	0.180
IBD	Periodontitis	Inverse variance weighted	0.989	0.898	1.088	0.813
IBD	Periodontitis	MR Egger	1.027	0.919	1.148	0.660
IBD	Periodontitis	Weighted median	1.005	0.915	1.103	0.917
CD	Periodontitis	Inverse variance weighted	0.945	0.830	1.077	0.398
CD	Periodontitis	MR Egger	1.000	0.864	1.156	0.996
CD	Periodontitis	Weighted median	0.967	0.864	1.082	0.558
UC	Periodontitis	Inverse variance weighted	1.040	0.928	1.165	0.504
UC	Periodontitis	MR Egger	1.075	0.930	1.242	0.385
UC	Periodontitis	Weighted median	1.084	0.957	1.228	0.205

*IBD* inflammatory bowel disease, *CD* Crohn’s disease, *UC* ulcerative colitis, *MR* Mendelian randomization, *OR* odds ratio, *CI* confidence interval, *P*: *P* value tests the null hypothesis of no association with exposure.

**Table 2:** Assessing heterogeneity through the inverse-variance weighted and MR Egger regression.

Outcome	Exposure	Method	Q	df	P-value
Periodontitis	IBD	Inverse variance weighted	133.112	152.000	0.863
Periodontitis	IBD	MR Egger	132.508	151.000	0.858
Periodontitis	CD	Inverse variance weighted	127.759	129.000	0.514
Periodontitis	CD	MR Egger	127.406	128.000	0.498
Periodontitis	UC	Inverse variance weighted	97.321	95.000	0.415
Periodontitis	UC	MR Egger	96.670	94.000	0.405
IBD	Periodontitis	Inverse variance weighted	8.553	5.000	0.128
IBD	Periodontitis	MR Egger	6.304	4.000	0.178
CD	Periodontitis	Inverse variance weighted	10.179	5.000	0.070
CD	Periodontitis	MR Egger	7.008	4.000	0.135
UC	Periodontitis	Inverse variance weighted	7.095	5.000	0.214
UC	Periodontitis	MR Egger	6.152	4.000	0.188

*IBD* inflammatory bowel disease, *CD* Crohn's disease, *UC* ulcerative colitis, *MR* Mendelian randomization,* Q* heterogeneity statistic *Q*, *df* degree of freedom, *P*: *P* value tests the null hypothesis of no association with exposure.

**Table 3:** Assessing directional pleiotropy through MR-Egger intercept.

Outcome	Exposure	Intercept	SE	*P*
Periodontitis	IBD	0.003	0.003	0.439
Periodontitis	CD	0.002	0.004	0.553
Periodontitis	UC	0.005	0.006	0.428
IBD	Periodontitis	− 0.016	0.013	0.298
CD	Periodontitis	− 0.024	0.018	0.250
UC	Periodontitis	− 0.013	0.017	0.478

*IBD* inflammatory bowel disease, *CD* Crohn's disease, *UC* ulcerative colitis, *MR* Mendelian randomization, *SE* standard error, *P*: *P* value tests the null hypothesis of no association with exposure.

### Causal effects of periodontitis on IBD and its subtypes

#### Character of selected SNPs

In the reverse direction, considering that no SNP was associated with periodontitis at the level of P < 5 × 10^−8^, the genetic instruments with P < 5 × 10^−6^ were selected. We identified 8 independent SNPs associated with periodontitis with *P* < 5 × 10^−6^. One of the genetic variants was palindromic and thus removed. The rest 7 SNPs were all available in the summary statistic of IBD and its subtypes. All these SNPs were independent in the LD clumping; their F-statistic values were all larger than 10. Another SNP was excluded after searching the PhenoScanner database. Finally, six SNPs remained as IVs of periodontitis; Tables S5–S7 present detailed information of these SNPs.

#### MR and sensitivity analysis

**Figure 3:**
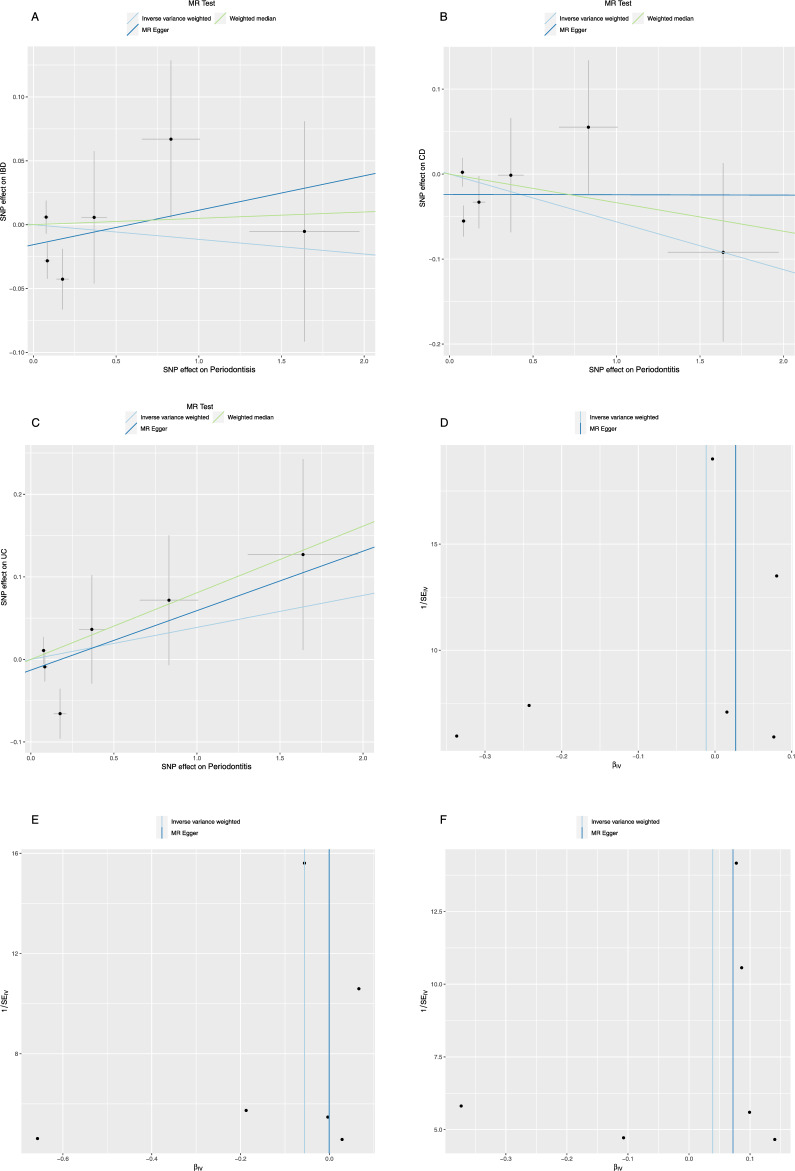
Scatter plot and Funnel plot of the causality of periodontitis on IBD, CD, and UC. (**A**) Scatter plot of the causal effect of periodontitis on IBD; (**B**) Scatter plot of the causal effect of periodontitis on CD; (**C**) Scatter plot of the causal effect of periodontitis on UC; (**D**) Funnel plot of the causal effect of periodontitis on IBD; (**E**) Funnel plot of the causal effect of periodontitis on CD; (**F**) Funnel plot of the causal effect of periodontitis on UC.

In the fundamental analysis, the IVW analysis, no statistically significant causal effects of genetically predicted periodontitis on IBD as a whole, CD and UC were found [periodontitis on IBD: odds ratio (OR) = 0.989, 95% CI = 0.898–1.088, *P* = 0.813; CD: OR = 0.945, 95% CI = 0.830–1.077, *P* = 0.398; UC: OR = 1.040, 95% CI = 0.928–1.165, *P* = 0.504] (Table 1, Fig. 3). The weighted median and MR Egger regression method showed the same results with point estimates slightly above and below 1 with highly overlapping CIs (Table 1, Fig. 3). No violations of the second and third assumptions were found after statistical analysis. No heterogeneity was noted between the individual SNP of periodontitis in the heterogeneity analysis (IBD: *P* value of IVW method: 0.128, *P* value of MR Egger method: 0.178; CD: *P* value of IVW method: 0.070, *P* value of MR Egger method: 0.135; UC: *P* value of IVW method: 0.214, *P* value of MR Egger method: 0.188) (Table 2). No horizontal pleiotropy was detected in the Egger intercept test (IBD: intercept = − 0.016, *P* = 0.298; CD: intercept = − 0.024, *P* = 0.250; UC: intercept = − 0.013, *P* = 0.478) (Table 3). The MR pleiotropy residual sum and outlier test outcomes demonstrated that there were no horizontal pleiotropic outliers. The “leave-one-out” test did not identify any leverage point with high influence. All sensitive analyses supported that there is no causal effect of genetically predicted periodontitis on IBD, UC, and CD. The funnel plots and leave-one-out analysis plots are shown in Fig. 3 and Figs. S4–S6. LHC-MR also demonstrated no causal effect of periodontitis on IBD as a whole and its subtypes (Periodontitis-IBD: SE = 0.358, *P* = 0.682; Periodontitis-UC: SE = 0.203, *P* = 0.944; Periodontitis-CD: SE = 0.319, *P* = 0.846).

## Discussion

This two-sample MR among people of European descent revealed that the genetic liability for IBD, CD, and UC did not affect the risk of periodontitis or vice versa. Subsequent sensitivity analyses did not detect any horizontal pleiotropy and heterogeneity, further revealing the reliability of these results.

The association between periodontitis and IBD has been shown but with insufficient causal evidence. A large-scale cross-sectional study reported that Chinese patients with IBD have a higher prevalence, severity, and extent of periodontitis than controls^9^; similar results were noted in several case–control studies^25,26^. However, a gap exists between causality and association. In concept, cross-sectional cannot infer causal associations because of the absence of chronological sequence and so do case control studies mainly because of the high occurrence recall bias. Although meta-analyses evidence is now available, there is still remarkable heterogeneity in the methods and results of the study of the association between the two diseases^27–29^. Furthermore, these meta-analyses mainly included case–control studies, making weak causal inferences. Population-based studies with a cohort design provide an approach to infer causality as the onset of the exposure can be observed to happen before or after the outcome of interest. A large-scale cohort study reported an increased prevalence of subsequent periodontitis among patients with CD^30^. Notably, this study was conducted among Asian individuals instead of European individuals, and the population stratification effect makes it hard to conclude that its outcome contradicts our results^31^. Additionally, confounders can hinder cohort studies and make the causal association of these two illnesses unpredictable. For example, patients with periodontitis or IBD commonly suffer from numerous comorbidities; the common pathways of these comorbidities potentially contribute to the association between periodontitis and IBD^32,33^. MR does provide an effective strategy to decipher causality while significantly weakening the interference of confounder. It is reasonable to believe our present MR study was more sufficiently powered to assess the causality between periodontitis and IBD. In conclusion, the available studies demonstrated weak evidence on the causality between periodontitis and IBD in the European population, whereas our MR, serving as a robust alternative, indicated the absence of causality between IBD and periodontitis.

The hypothesized mechanism underlying the association of IBD and periodontitis remains elusive; the available evidence is insufficient to contradict the results of this two-sample MR. A current hypothesis of the association suggested an oral-gut interaction depending on the immune cell activation and microbiome to synergistically promote inflammation in genetically susceptible individuals^34^. However, no study on humans has conclusively proved this hypothesis yet. Some proposed pathways, such as calprotectin, are not specific and shared by several chronic inflammatory conditions with neutrophil infiltration^30^. These shared inflammatory pathways of these systemic diseases may confound the causal association and lead to discrepancies in outcomes between the previous observational studies and the present MR. Moreover, it is still unclear whether inflammatory processes in various peripheral tissues, such as periodontitis and IBD, are interlinked via inflammatory adaptations of the bone marrow, a central hub^35^. In summary, the causality between periodontitis and IBD has not been conclusively demonstrated at mechanism level; there is no reason to reject the results of this MR.

Interestingly, a recently published MR regarding IBD and periodontitis shows some different results to this study^36^. It concludes that IBD as a whole and UC have causal effect on periodontitis. In the reverse direction, periodontitis has an association with IBD and CD. This difference might root from the selection of datasets. Its GWAS of IBD and periodontitis both differ from ours in the aspect of sample size and population source. Specifically, the GWAS of IBD we use is a collective one, not only totally include GWAS used in the published MR, but also have additional new GWAS data^18^. The GWAS summary statistics of periodontitis we apply have five times more cases than their. Furthermore, both the two MR target European population. In their study, a single population source (Finland) of GWAS is employed to represent European population, but our GWAS of periodontitis has multiple population source (UK, U.S., Germany and Sweden). A detailed comparation of the GWAS summary statistics in the two studies is presented as a table (Table S8). Sample size and representativeness are key factors in determining the statistic power of MR outcomes^13^. Our GWAS, with larger sample size and multiple population sources can increase the statistic power of MR. In terms of the analysis methods, both studies apply almost the same methods including IVW, MR pleiotropy residual sum, outlier test, Weighted median and MR Egger regression. However, to further strengthen our analysis, we use LHC-MR as an additional sensitivity analyses^24^. This method appropriately uses genome-wide genetic markers to estimate bidirectional causal effects, direct heritability, confounder effect and population stratification while accounting for sample overlap. LHC-MR extends the standard two-sample MR by modeling a latent heritable confounder that affects both the exposure and outcome traits. It can differentiate SNPs based on their co-association to a pair of traits. Thus, the unbiased bidirectional causal effect between these two traits is estimated simultaneously as well as the confounder effect on each trait. According to LHC-MR, no bidirectional causal association exists in both directions, which further ensure the reliability of our result, make our outcome less prone to weak instrument bias and endow this MR with substantially high statistical power. In summary, the selection of GWAS leads to the difference of the two MR results in a large extent and more new GWAS and novel MR approaches are awaited for more authentic association between IBD and periodontitis.

The present study has four main strengths. First, in this two-sample MR, large-scale GWAS datasets from two independent populations of European descent were used to ensure sufficient statistical power for inferring a causal association and minimizing the effect of population stratification. Second, the high degree of consistency from various applied complementary MR approaches proved the stability of our study. Third, multiple sensitivity analyses showed no heterogeneity and pleiotropy and further supported our confidence in the established associations. Fourth, with the advantage of the MR study design, reverse causality could be eliminated and as did most potential confounding effects.

Nonetheless, there are several limitations to this study. First, the results were only drawn from the European population; thus, caution should be exerted when extrapolating the results to other ethnicities and races. Future MR studies need to incorporate additional genetic instruments thereby increasing the proportion of IBD and periodontitis explained. Second, given any genetic variant only explains a proportion of phenotypic variance, future MR studies should include larger samples and more valid IVs to make a robust causal inference. Third, these MR findings only reflected the change in outcomes due to a genetically predisposed (lifetime) status of exposures; the short-term effect of exposures on outcomes was unknown, highlighting the discrepancy between the results of this MR and the previous observational studies.

In conclusion, our two-sample MR study did not show a causal effect of genetically proxied IBD as an exposure on the development of periodontitis as an outcome using the summary statistics data within individuals of European ancestry. Similarly, in the reverse inference, limited evidence supported a causal role of genetic liability for periodontitis on IBD. Our results can provide a reference for future large-scale RCTs and might predict the results of these RCTs. However, caution should be exerted when it comes to clinical relevance and further studies are needed to clarify the relationship between IBD and periodontitis.

### Supplementary Information

Supplementary Information.

## Data Availability

The periodontitis summary statistic data are available at https://data.bris.ac.uk/data/dataset/. The IBD, CD, and UC summary statistic data can be accessed at https://www.ebi.ac.uk/gwas/studies/GCST004131, https://www.ebi.ac.uk/gwas/studies/GCST004132, https://www.ebi.ac.uk/gwas/studies/GCST004133.

## Abbreviations

CD: Crohn’s disease
CI: Confidence intervals
GLIDE: Gene-Lifestyle Interactions in Dental Endpoints
GWAS: Genome-wide association study
IBD: Inflammatory bowel disease
IV: Instrumental variable
LD: Linkage disequilibrium
MR: Mendelian randomization
OR: Odds ratio
RCT: Randomized controlled trial
SNP: Single nucleotide polymorphism
STROBE: Strengthening the Reporting of Observational Studies in Epidemiology
UC: Ulcerative colitis
