# Supplementary material for: Deciphering genetic causality between inflammatory bowel disease and periodontitis through bi-directional two-sample Mendelian randomization
Source: Sci Rep. 2023 Oct 30;13:18620. doi: 10.1038/s41598-023-45527-z (PMC10616190; doi:10.1038/s41598-023-45527-z)
Supplement: Supplementary file 1 — Supplementary Information. [file 41598_2023_45527_MOESM1_ESM.docx]

**Deciphering genetic causality between inflammatory bowel disease and periodontitis through bi-directional two-sample Mendelian randomization**

Feiyan Yu,^1,2,#^ Yang Yang, ^1,2,#^ Dongchao Wu, ^1,2^ Minjing Chang, ^3^ Chong Han, ^1,2^ Qianqian Wang, ^1,2^ Yi Li, ^2^ Dongning He, ^1,2,^*

1. Shanxi Medical University School and Hospital of Stomatology, Taiyuan, China;

2. Shanxi Province Key Laboratory of Oral Diseases Prevention and New Materials, Taiyuan, China;

3. Shanxi Key Laboratory of Big Data for Clinical Decision, Shanxi Medical University, Taiyuan, China;

^#^These authors contributed equally to this work.

***Correspondence to**

Dongning He, Department of Implantology, Shanxi Medical University School and Hospital of Stomatology, No. 63, New South Road, Yingze District, Taiyuan, Shanxi 030001, P.R. China, Tel: +86-0351-4690227, fax: +86-0351-4690227 Email: hdn00@163.com.

**Supplementary Figures**

Supplementary Figure 1. Leave‑one‑out analysis of the causal effect of IBD on periodontitis.

Supplementary Figure 2. Leave‑one‑out analysis of the causal effect of CD on periodontitis.

Supplementary Figure 3. Leave‑one‑out analysis of the causal effect of UC on periodontitis.

Supplementary Figure 4. Leave‑one‑out analysis of the causal effect of periodontitis on IBD.

Supplementary Figure 5. Leave‑one‑out analysis of the causal effect of periodontitis on CD.

Supplementary Figure 6. Leave‑one‑out analysis of the causal effect of periodontitis on UC.

**Supplementary Tables**

Supplementary Table 1. Characteristics of studies used for primary MR analysis.

Supplementary Table 2. Characteristics of the SNPs used as IVs in the causal association of IBD with periodontitis.

Supplementary Table 3. Characteristics of the SNPs used as IVs in the causal association of CD with periodontitis.

Supplementary Table 4. Characteristics of the SNPs used as IVs in the causal association of UC with periodontitis.

Supplementary Table 5. Characteristics of the SNPs used as IVs in the causal association of periodontitis of IBD.

Supplementary Table 6. Characteristics of the SNPs used as IVs in the causal association of periodontitis of CD.

Supplementary Table 7. Characteristics of the SNPs used as IVs in the causal association of periodontitis of UC.

Supplementary Table 8. GWAS summary statistics comparation of two MR.

**Supplementary Figures**

**
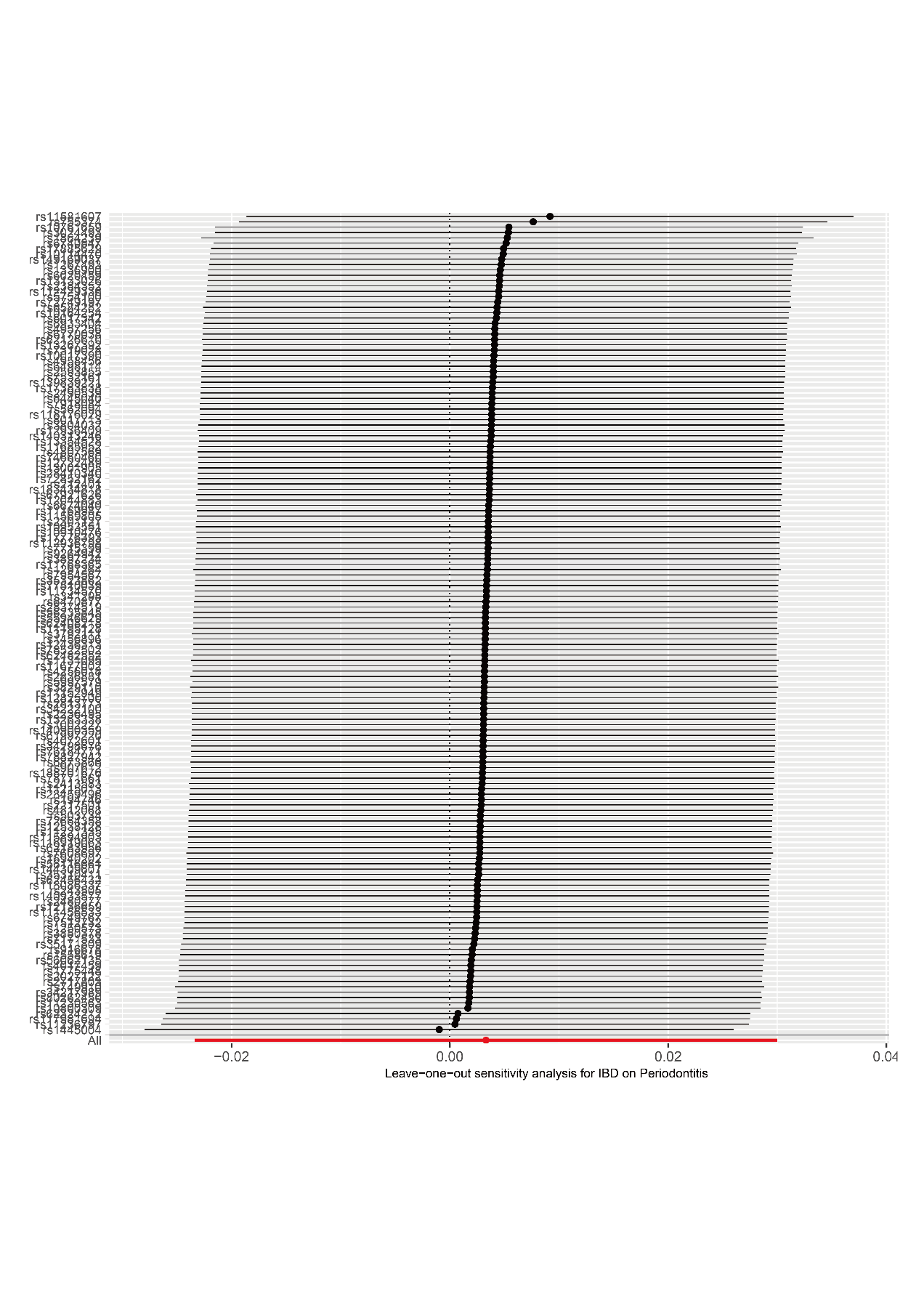
**

**Supplementary Figure 1. Leave‑one‑out analysis of the causal effect of IBD on periodontitis.**


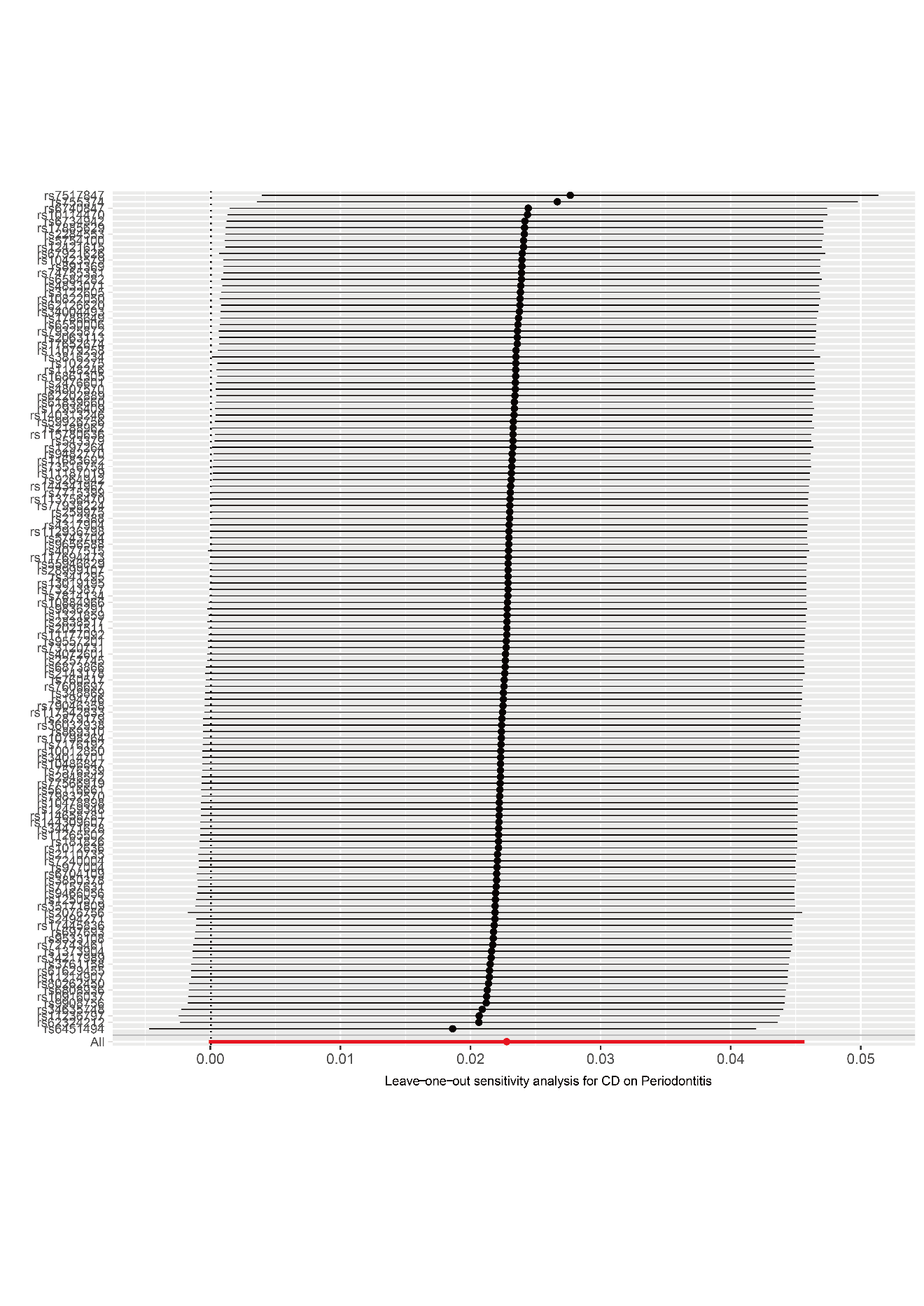


**Supplementary Figure 2. Leave‑one‑out analysis of the causal effect of CD on periodontitis.**


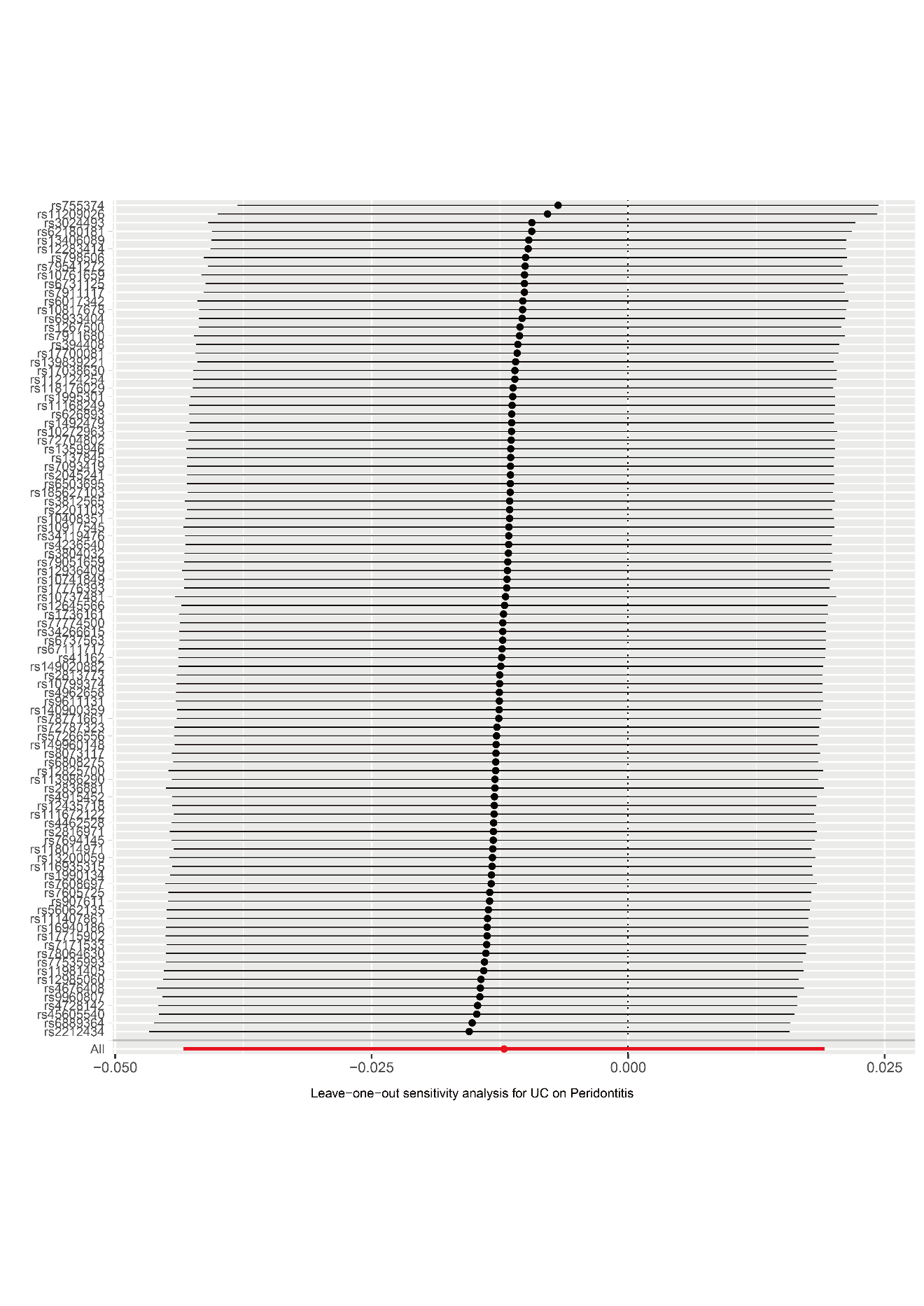


**Supplementary Figure 3. Leave‑one‑out analysis of the causal effect of UC on periodontitis.**


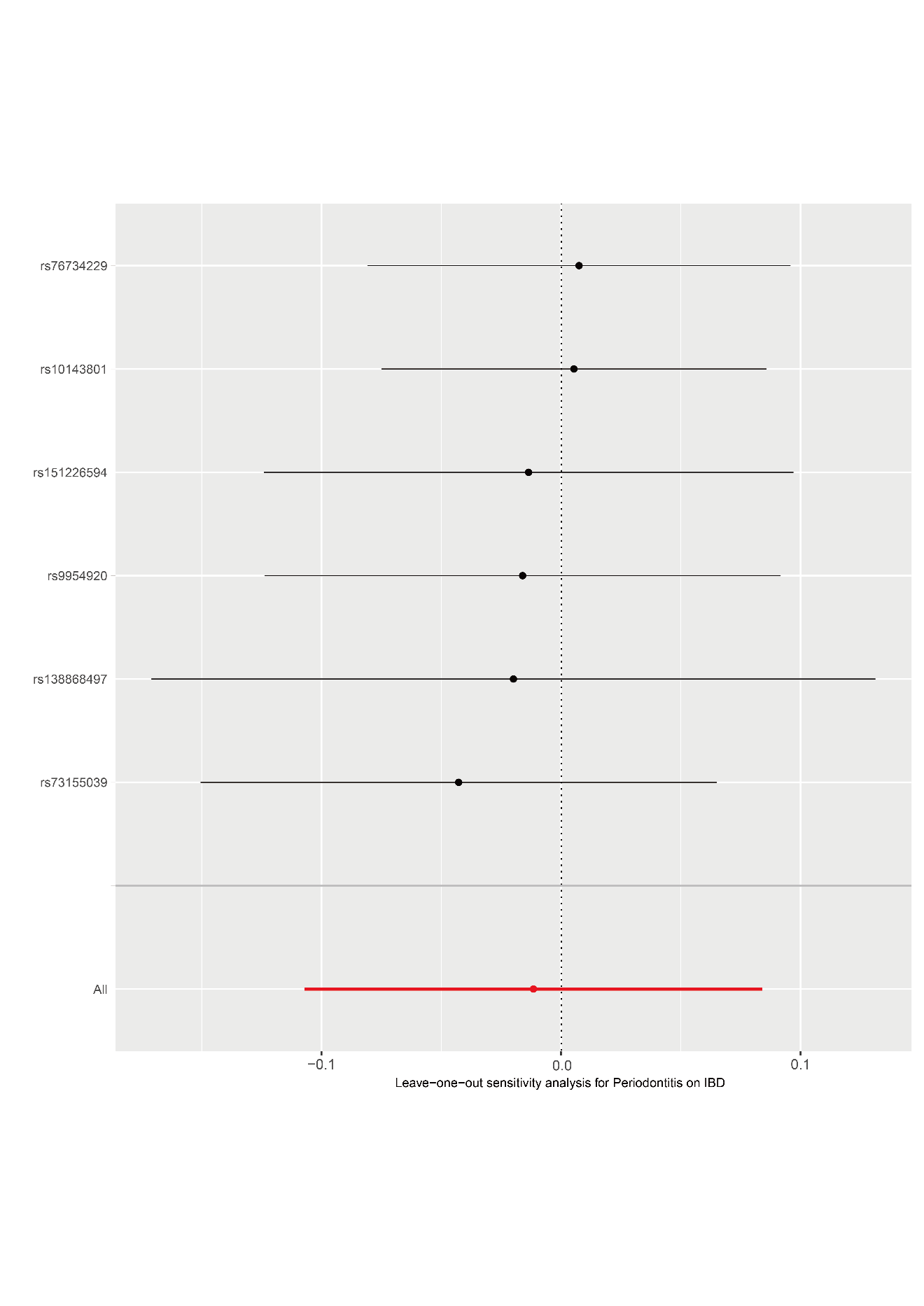


**Supplementary Figure 4. Leave‑one‑out analysis of the causal effect of periodontitis on IBD.**


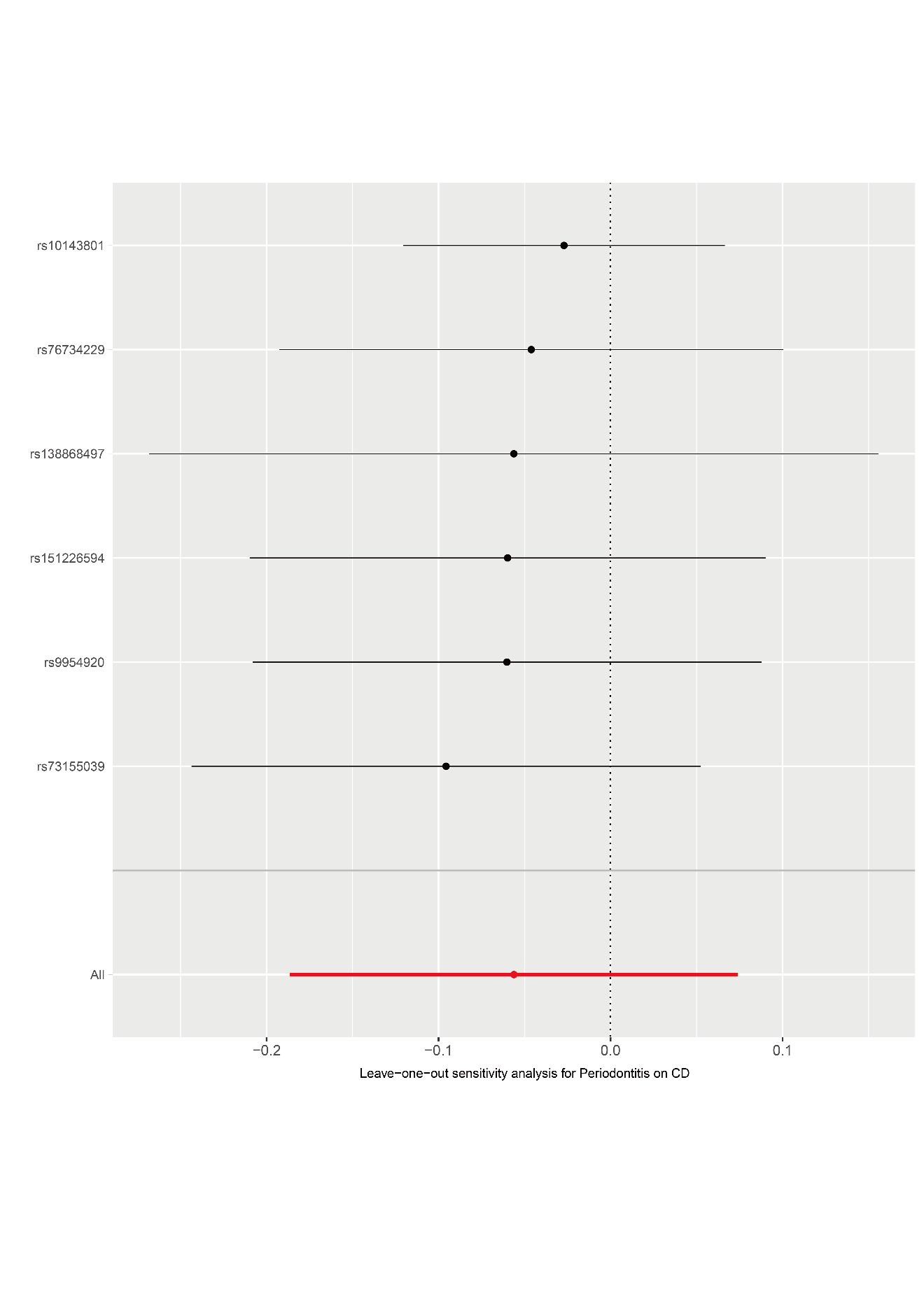


**Supplementary Figure 5. Leave‑one‑out analysis of the causal effect of periodontitis on CD.**


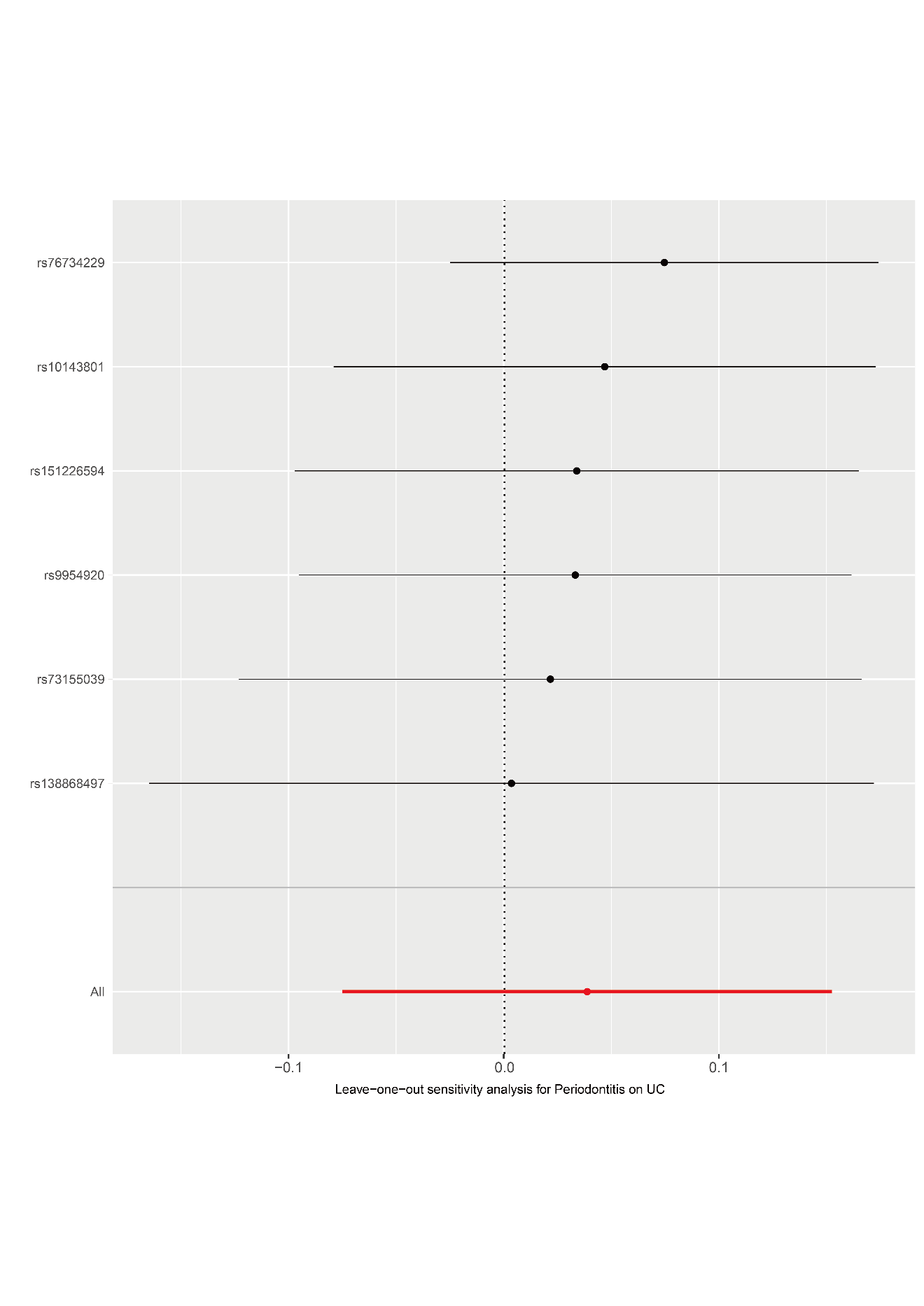


**Supplementary Figure 6. Leave‑one‑out analysis of the causal effect of periodontitis on UC.**

**Supplementary Table 1. Characteristics of studies used for primary MR analysis.**

| Traits | Consortium | Cases | Control | Sample size | Year | Population | PMID |
| --- | --- | --- | --- | --- | --- | --- | --- |
| IBD (de Lange et al., 2017) | NA | 25042 | 34915 | 59957 | 2017 | European | 28067908 |
| CD (de Lange et al., 2017) | NA | 12194 | 28072 | 40266 | 2017 | European | 28067908 |
| UC (de Lange et al., 2017) | NA | 12366 | 33609 | 45975 | 2017 | European | 28067908 |
| Periodontitis (Shungin et al., 2019) | GLIDE | 17353 | 28210 | 45563 | 2019 | European | 31235808 |

Note: IBD, inflammatory bowel disease; CD, Crohn's disease; UC, ulcerative colitis; NA, not available; GLIDE, Gene-Lifestyle Interactions and Dental Endpoints;

de Lange, K. M., Moutsianas, L., Lee, J. C., Lamb, C. A., Luo, Y., Kennedy, N. A., … Barrett, J. C. (2017). Genome-wide association study implicates immune activation of multiple integrin genes in inflammatory bowel disease. *Nature Genetics*, *49*(2), 256–261. doi: 10.1038/ng.3760

Shungin, D., Haworth, S., Divaris, K., Agler, C. S., Kamatani, Y., Keun Lee, M., … Johansson, I. (2019). Genome-wide analysis of dental caries and periodontitis combining clinical and self-reported data. *Nature Communications*, *10*(1), 2773. doi: 10.1038/s41467-019-10630-1

**Supplementary Table 2. Characteristics of the SNPs used as IVs in the causal association of IBD with periodontitis.**

| SNP | Chr | Pos | EA | OA | EAF | Association with IBD | | |  | Association with periodontitis | | |
| --- | --- | --- | --- | --- | --- | --- | --- | --- | --- | --- | --- | --- |
|  |  |  |  |  |  | BETA | SE | *P* |  | BETA | SE | *P* |
| rs10017390 | 4 | 94882341 | A | G | NA | -0.059 | 0.013 | 3.83E-06 |  | 0.295 | 0.017 | 0.016 |
| rs1002227 | 11 | 17405347 | A | C | NA | 0.067 | 0.014 | 7.30E-07 |  | 0.735 | 0.006 | 0.018 |
| rs10114470 | 9 | 117547772 | C | T | NA | 0.148 | 0.014 | 4.10E-27 |  | 0.340 | -0.016 | 0.017 |
| rs10164254 | 18 | 77766322 | G | T | NA | 0.069 | 0.015 | 2.52E-06 |  | 0.169 | -0.025 | 0.018 |
| rs10746475 | 1 | 8168261 | A | T | NA | 0.131 | 0.016 | 1.58E-15 |  | 0.039 | -0.039 | 0.019 |
| rs10761659 | 10 | 64445564 | G | A | NA | 0.159 | 0.013 | 2.30E-36 |  | 0.278 | -0.017 | 0.016 |
| rs10800309 | 1 | 161472158 | G | A | NA | -0.123 | 0.013 | 1.94E-20 |  | 0.238 | -0.019 | 0.016 |
| rs10910476 | 1 | 234734956 | T | C | NA | 0.069 | 0.013 | 8.37E-08 |  | 0.802 | -0.004 | 0.017 |
| rs10953551 | 7 | 107480901 | G | A | NA | -0.103 | 0.013 | 4.94E-16 |  | 0.878 | 0.003 | 0.016 |
| rs111456533 | 10 | 126439381 | A | G | NA | -0.103 | 0.017 | 1.18E-09 |  | 0.370 | -0.018 | 0.020 |
| rs11152949 | 6 | 106449085 | G | A | NA | 0.102 | 0.013 | 1.56E-14 |  | 0.860 | 0.003 | 0.018 |
| rs11169987 | 12 | 52429494 | G | A | NA | -0.120 | 0.024 | 7.21E-07 |  | 0.768 | 0.009 | 0.029 |
| rs11195128 | 10 | 112186148 | T | C | NA | 0.079 | 0.013 | 2.74E-09 |  | 0.938 | 0.001 | 0.017 |
| rs11215013 | 11 | 114356009 | T | C | NA | 0.253 | 0.054 | 2.36E-06 |  | 0.513 | 0.050 | 0.077 |
| rs11221335 | 11 | 128385906 | C | T | NA | 0.083 | 0.015 | 2.44E-08 |  | 0.492 | 0.013 | 0.019 |
| rs11230563 | 11 | 60776209 | T | C | NA | -0.062 | 0.013 | 1.95E-06 |  | 0.025 | -0.036 | 0.016 |
| rs11236797 | 11 | 76299649 | A | C | NA | 0.149 | 0.013 | 7.19E-33 |  | 0.109 | 0.025 | 0.016 |
| rs112429336 | 11 | 65881627 | T | C | NA | 0.113 | 0.023 | 6.61E-07 |  | 0.087 | -0.053 | 0.031 |
| rs112936798 | 1 | 39802381 | C | A | NA | -0.184 | 0.033 | 2.89E-08 |  | 0.624 | 0.043 | 0.087 |
| rs1131095 | 3 | 49714225 | C | T | NA | 0.164 | 0.013 | 1.22E-35 |  | 0.928 | 0.002 | 0.017 |
| rs113580223 | 3 | 56932367 | T | A | NA | -0.155 | 0.033 | 3.08E-06 |  | 0.771 | 0.016 | 0.056 |
| rs11569805 | 1 | 12137603 | C | A | NA | -0.067 | 0.014 | 1.76E-06 |  | 0.668 | 0.010 | 0.024 |
| rs11581607 | 1 | 67707690 | A | G | NA | -0.658 | 0.029 | ######## |  | 0.177 | 0.043 | 0.032 |
| rs115894903 | 5 | 75973742 | T | C | NA | 0.100 | 0.020 | 3.11E-07 |  | 0.450 | 0.019 | 0.025 |
| rs11637301 | 15 | 61047898 | T | A | NA | -0.091 | 0.018 | 6.60E-07 |  | 0.855 | -0.004 | 0.022 |
| rs11677002 | 2 | 28614401 | C | T | NA | -0.093 | 0.013 | 1.37E-13 |  | 0.896 | -0.002 | 0.016 |
| rs11685952 | 2 | 161410412 | T | G | NA | -0.071 | 0.015 | 2.28E-06 |  | 0.599 | 0.009 | 0.018 |
| rs116919063 | 12 | 92843430 | A | G | NA | 0.240 | 0.052 | 3.72E-06 |  | 0.259 | 0.100 | 0.089 |
| rs11734570 | 4 | 38588453 | A | G | NA | 0.069 | 0.013 | 4.80E-08 |  | 0.971 | -6.00E-04 | 0.016 |
| rs11739135 | 5 | 131733397 | C | G | NA | 0.137 | 0.013 | 1.10E-27 |  | 0.959 | -8.00E-04 | 0.016 |
| rs11768365 | 7 | 6545188 | G | A | NA | -0.084 | 0.015 | 3.88E-08 |  | 0.876 | 0.003 | 0.018 |
| rs117981694 | 12 | 40822098 | A | G | NA | 0.345 | 0.041 | 4.53E-17 |  | 0.109 | 0.061 | 0.038 |
| rs118086337 | 18 | 33426894 | T | G | NA | -0.102 | 0.021 | 1.87E-06 |  | 0.198 | -0.039 | 0.030 |
| rs118176029 | 12 | 21336573 | C | T | NA | -0.208 | 0.044 | 2.83E-06 |  | 0.220 | 0.113 | 0.092 |
| rs12044883 | 1 | 93820575 | A | G | NA | 0.061 | 0.013 | 2.26E-06 |  | 0.690 | -0.006 | 0.016 |
| rs12136659 | 1 | 172844250 | C | T | NA | 0.087 | 0.014 | 1.02E-09 |  | 0.355 | 0.017 | 0.018 |
| rs12436313 | 14 | 105509242 | G | A | NA | 0.074 | 0.015 | 1.38E-06 |  | 0.907 | 0.002 | 0.018 |
| rs1250573 | 10 | 81042475 | A | G | NA | -0.098 | 0.014 | 1.11E-12 |  | 0.353 | -0.016 | 0.018 |
| rs12538128 | 7 | 36001017 | G | A | NA | -0.069 | 0.014 | 5.35E-07 |  | 0.442 | -0.014 | 0.019 |
| rs1267493 | 6 | 14716354 | C | T | NA | 0.100 | 0.016 | 2.77E-10 |  | 0.160 | -0.027 | 0.019 |
| rs12722489 | 10 | 6102012 | T | C | NA | -0.091 | 0.017 | 1.28E-07 |  | 0.639 | 0.011 | 0.022 |
| rs12825700 | 12 | 68492980 | A | G | NA | 0.132 | 0.013 | 1.27E-25 |  | 0.882 | 0.003 | 0.017 |
| rs12936409 | 17 | 38043649 | T | C | NA | 0.141 | 0.012 | 7.73E-30 |  | 0.801 | -0.004 | 0.016 |
| rs1297264 | 21 | 16816017 | G | A | NA | -0.146 | 0.013 | 3.98E-31 |  | 0.970 | 6.00E-04 | 0.016 |
| rs12984626 | 19 | 47140080 | C | G | NA | -0.075 | 0.015 | 3.02E-07 |  | 0.085 | -0.031 | 0.018 |
| rs13001905 | 2 | 242476987 | G | A | NA | -0.067 | 0.014 | 9.12E-07 |  | 0.630 | 0.009 | 0.018 |
| rs13103812 | 4 | 36078004 | G | C | NA | -0.065 | 0.012 | 1.74E-07 |  | 0.862 | 0.003 | 0.016 |
| rs13133026 | 4 | 187045817 | C | T | NA | -0.059 | 0.013 | 3.39E-06 |  | 0.065 | 0.030 | 0.017 |
| rs1319951 | 18 | 46404813 | G | C | NA | -0.085 | 0.015 | 7.50E-09 |  | 0.155 | -0.026 | 0.019 |
| rs13263338 | 8 | 49418561 | C | T | NA | -0.074 | 0.014 | 2.66E-07 |  | 0.778 | -0.005 | 0.017 |
| rs13267392 | 8 | 145245647 | T | C | NA | 0.084 | 0.016 | 1.58E-07 |  | 0.178 | -0.036 | 0.027 |
| rs13354528 | 5 | 141476769 | T | C | NA | 0.082 | 0.015 | 9.22E-08 |  | 0.592 | -0.010 | 0.019 |
| rs1336900 | 1 | 150679033 | A | G | NA | -0.085 | 0.013 | 2.98E-11 |  | 0.203 | 0.020 | 0.016 |
| rs139839221 | 4 | 84230033 | T | C | NA | 0.238 | 0.049 | 1.39E-06 |  | 0.091 | -0.205 | 0.121 |
| rs140152928 | 2 | 132143532 | A | T | NA | -0.266 | 0.052 | 3.60E-07 |  | 0.077 | 0.290 | 0.164 |
| rs140313246 | 17 | 7007622 | A | G | NA | -0.136 | 0.027 | 3.26E-07 |  | 0.585 | 0.016 | 0.030 |
| rs140900359 | 6 | 69009744 | C | T | NA | 0.264 | 0.056 | 2.42E-06 |  | 0.580 | 0.064 | 0.116 |
| rs140933577 | 13 | 40836270 | C | T | NA | -0.186 | 0.031 | 1.13E-09 |  | 0.369 | -0.035 | 0.039 |
| rs144309607 | 19 | 10492274 | T | C | NA | -0.251 | 0.037 | 7.68E-12 |  | 0.428 | -0.043 | 0.054 |
| rs1445004 | 5 | 40414419 | T | C | NA | 0.169 | 0.013 | 3.48E-40 |  | 0.033 | 0.034 | 0.016 |
| rs1456896 | 7 | 50304461 | T | C | NA | 0.088 | 0.013 | 4.50E-11 |  | 0.928 | 0.002 | 0.017 |
| rs149169037 | 7 | 20577298 | A | G | NA | -0.134 | 0.024 | 3.26E-08 |  | 0.077 | 0.054 | 0.031 |
| rs1555401 | 6 | 117767335 | C | G | NA | 0.065 | 0.013 | 6.09E-07 |  | 0.135 | -0.025 | 0.017 |
| rs1558619 | 2 | 102931550 | T | G | NA | -0.084 | 0.012 | 8.90E-12 |  | 0.204 | -0.020 | 0.016 |
| rs16940202 | 16 | 86014241 | C | T | NA | 0.113 | 0.017 | 2.50E-11 |  | 0.459 | 0.019 | 0.025 |
| rs17383838 | 8 | 102258491 | T | C | NA | -0.229 | 0.050 | 4.63E-06 |  | 0.209 | 0.114 | 0.091 |
| rs1775448 | 1 | 197615130 | G | A | NA | -0.085 | 0.013 | 9.82E-11 |  | 0.137 | -0.025 | 0.017 |
| rs17776393 | 14 | 50574019 | A | G | NA | -0.301 | 0.059 | 3.24E-07 |  | 0.756 | 0.028 | 0.090 |
| rs17885629 | 17 | 40525098 | G | A | NA | -0.110 | 0.013 | 3.95E-18 |  | 0.212 | 0.020 | 0.016 |
| rs183434818 | 4 | 144921234 | G | A | NA | -0.101 | 0.020 | 6.57E-07 |  | 0.369 | 0.049 | 0.054 |
| rs1864239 | 15 | 80205354 | G | A | NA | 1.337 | 0.178 | 6.27E-14 |  | 0.730 | -0.020 | 0.059 |
| rs188701676 | 7 | 75154838 | A | G | NA | -0.364 | 0.077 | 2.00E-06 |  | 0.504 | -0.092 | 0.137 |
| rs1887428 | 9 | 4984530 | C | G | NA | -0.164 | 0.013 | 2.46E-36 |  | 0.844 | 0.003 | 0.016 |
| rs194746 | 14 | 69282887 | T | C | NA | 0.083 | 0.012 | 1.84E-11 |  | 0.661 | 0.007 | 0.016 |
| rs2027122 | 9 | 27138214 | G | A | NA | -0.071 | 0.015 | 1.21E-06 |  | 0.052 | -0.035 | 0.018 |
| rs212401 | 6 | 159472682 | G | A | NA | -0.072 | 0.014 | 1.04E-07 |  | 0.680 | 0.007 | 0.018 |
| rs2217501 | 12 | 70848295 | C | T | NA | -0.064 | 0.014 | 4.97E-06 |  | 0.526 | -0.011 | 0.017 |
| rs2236495 | 9 | 4844214 | C | T | NA | -0.075 | 0.016 | 1.52E-06 |  | 0.766 | -0.006 | 0.019 |
| rs2301127 | 16 | 11367477 | A | G | NA | 0.078 | 0.013 | 4.96E-10 |  | 0.828 | -0.004 | 0.016 |
| rs2384352 | 10 | 35492832 | G | A | NA | 0.095 | 0.013 | 3.12E-13 |  | 0.271 | -0.018 | 0.016 |
| rs2413583 | 22 | 39659773 | T | C | NA | -0.173 | 0.017 | 4.60E-24 |  | 0.797 | -0.005 | 0.021 |
| rs243505 | 7 | 148435339 | G | A | NA | -0.081 | 0.013 | 3.04E-10 |  | 0.393 | -0.014 | 0.016 |
| rs2480277 | 10 | 30703513 | C | A | NA | 0.090 | 0.013 | 1.36E-12 |  | 0.422 | 0.013 | 0.016 |
| rs2490539 | 1 | 1882545 | A | G | NA | -0.063 | 0.014 | 2.94E-06 |  | 0.373 | 0.017 | 0.019 |
| rs2593855 | 3 | 71175495 | T | C | NA | -0.083 | 0.014 | 2.54E-09 |  | 0.436 | 0.014 | 0.018 |
| rs2675670 | 10 | 75655628 | C | G | NA | 0.064 | 0.013 | 2.95E-07 |  | 0.483 | -0.011 | 0.016 |
| rs2717803 | 7 | 148211140 | T | G | NA | -0.060 | 0.013 | 3.63E-06 |  | 0.035 | -0.034 | 0.016 |
| rs2813773 | 1 | 110364821 | A | G | NA | 0.062 | 0.013 | 3.45E-06 |  | 0.764 | 0.005 | 0.016 |
| rs2836881 | 21 | 40466299 | T | G | NA | -0.164 | 0.015 | 1.96E-29 |  | 0.910 | -0.002 | 0.017 |
| rs28374519 | 16 | 28489342 | A | G | NA | -0.111 | 0.014 | 6.55E-16 |  | 0.977 | -7.00E-04 | 0.024 |
| rs28410340 | 7 | 70942107 | A | C | NA | -0.092 | 0.019 | 1.48E-06 |  | 0.674 | 0.009 | 0.022 |
| rs28459496 | 8 | 64849601 | T | C | NA | 0.105 | 0.022 | 1.74E-06 |  | 0.519 | 0.019 | 0.029 |
| rs28722705 | 5 | 55453942 | T | A | NA | -0.095 | 0.018 | 1.30E-07 |  | 0.111 | 0.037 | 0.023 |
| rs3024493 | 1 | 206943968 | A | C | NA | 0.191 | 0.017 | 4.04E-31 |  | 0.212 | -0.028 | 0.022 |
| rs341295 | 5 | 111848890 | T | C | NA | 0.070 | 0.012 | 1.45E-08 |  | 0.973 | -5.00E-04 | 0.016 |
| rs34217989 | 5 | 158564207 | C | T | NA | -0.079 | 0.014 | 1.80E-08 |  | 0.062 | -0.034 | 0.018 |
| rs34222100 | 16 | 50365136 | T | C | NA | -0.102 | 0.016 | 2.60E-10 |  | 0.824 | -0.004 | 0.019 |
| rs34298676 | 2 | 163126317 | G | A | NA | -0.140 | 0.028 | 7.56E-07 |  | 0.717 | -0.013 | 0.035 |
| rs35171809 | 6 | 167432766 | G | A | NA | 0.109 | 0.012 | 1.16E-18 |  | 0.383 | 0.014 | 0.016 |
| rs35310411 | 16 | 67385250 | T | C | NA | -0.183 | 0.039 | 2.53E-06 |  | 0.319 | -0.050 | 0.050 |
| rs35323862 | 6 | 127428232 | T | C | NA | 0.068 | 0.015 | 4.59E-06 |  | 0.914 | -0.002 | 0.019 |
| rs3792111 | 2 | 234179690 | T | C | NA | 0.139 | 0.012 | 5.12E-29 |  | 0.952 | 0.001 | 0.016 |
| rs3804032 | 21 | 45629691 | G | A | NA | -0.118 | 0.013 | 5.04E-21 |  | 0.736 | 0.005 | 0.016 |
| rs3829110 | 9 | 139269198 | G | A | NA | 0.157 | 0.013 | 3.52E-36 |  | 0.902 | 0.002 | 0.016 |
| rs3850378 | 14 | 88417517 | C | T | NA | 0.154 | 0.021 | 1.10E-13 |  | 0.371 | 0.023 | 0.025 |
| rs3897234 | 13 | 27542030 | C | T | NA | 0.097 | 0.015 | 1.90E-11 |  | 0.883 | -0.003 | 0.018 |
| rs4017459 | 1 | 160838953 | G | A | NA | 0.071 | 0.014 | 1.94E-07 |  | 0.075 | 0.030 | 0.017 |
| rs4072601 | 17 | 70641327 | A | G | NA | -0.126 | 0.018 | 8.43E-13 |  | 0.804 | -0.005 | 0.021 |
| rs4256018 | 20 | 6093889 | G | T | NA | 0.079 | 0.014 | 1.23E-08 |  | 0.866 | 0.003 | 0.017 |
| rs4712528 | 6 | 20678430 | C | G | NA | 0.104 | 0.015 | 7.14E-12 |  | 0.471 | 0.013 | 0.019 |
| rs4775717 | 15 | 48082196 | G | C | NA | -0.064 | 0.014 | 5.00E-06 |  | 0.898 | 0.002 | 0.018 |
| rs4807569 | 19 | 1123378 | C | A | NA | 0.128 | 0.015 | 4.24E-17 |  | 0.795 | -0.005 | 0.018 |
| rs4812068 | 20 | 57843903 | C | T | NA | -0.083 | 0.014 | 7.59E-10 |  | 0.603 | -0.009 | 0.016 |
| rs4957256 | 5 | 40207142 | T | C | NA | -0.118 | 0.016 | 3.37E-14 |  | 0.499 | 0.013 | 0.019 |
| rs4958456 | 5 | 149623365 | T | C | NA | -0.090 | 0.018 | 9.30E-07 |  | 0.352 | 0.021 | 0.023 |
| rs4971079 | 1 | 155130391 | A | G | NA | 0.075 | 0.012 | 1.32E-09 |  | 0.172 | 0.021 | 0.016 |
| rs503734 | 3 | 101023748 | G | A | NA | -0.069 | 0.012 | 2.67E-08 |  | 0.524 | -0.010 | 0.016 |
| rs55946629 | 2 | 43851246 | A | C | NA | 0.130 | 0.018 | 5.45E-13 |  | 0.946 | 0.002 | 0.023 |
| rs56062135 | 15 | 67455630 | T | C | NA | 0.138 | 0.015 | 1.37E-21 |  | 0.317 | 0.019 | 0.019 |
| rs56116661 | 3 | 188401160 | T | C | NA | -0.100 | 0.016 | 9.27E-10 |  | 0.468 | -0.015 | 0.020 |
| rs562094 | 5 | 172327361 | C | T | NA | -0.065 | 0.014 | 1.93E-06 |  | 0.466 | 0.012 | 0.017 |
| rs56235845 | 5 | 176798040 | G | T | NA | 0.088 | 0.014 | 1.77E-10 |  | 0.950 | 0.001 | 0.018 |
| rs5754100 | 22 | 21916166 | C | T | NA | 0.129 | 0.016 | 7.14E-16 |  | 0.308 | -0.022 | 0.022 |
| rs599400 | 18 | 2716496 | C | G | NA | 0.065 | 0.013 | 2.26E-07 |  | 0.732 | -0.005 | 0.016 |
| rs5997579 | 22 | 30537526 | C | T | NA | 0.069 | 0.012 | 2.63E-08 |  | 0.850 | 0.003 | 0.015 |
| rs6017342 | 20 | 43065028 | C | A | NA | 0.116 | 0.014 | 1.07E-17 |  | 0.403 | -0.016 | 0.019 |
| rs6020459 | 20 | 48960546 | C | T | NA | -0.069 | 0.014 | 1.02E-06 |  | 0.096 | 0.028 | 0.017 |
| rs6062496 | 20 | 62329099 | A | G | NA | 0.137 | 0.013 | 2.83E-26 |  | 0.238 | 0.020 | 0.017 |
| rs6087887 | 20 | 30950471 | T | A | NA | -0.061 | 0.013 | 9.69E-07 |  | 0.265 | -0.018 | 0.016 |
| rs61997220 | 6 | 149783095 | C | T | NA | -0.139 | 0.029 | 1.31E-06 |  | 0.667 | -0.018 | 0.043 |
| rs62126610 | 19 | 33748183 | G | A | NA | 0.141 | 0.017 | 2.60E-17 |  | 0.539 | -0.013 | 0.021 |
| rs62180107 | 2 | 199629721 | C | G | NA | -0.080 | 0.013 | 1.55E-09 |  | 0.937 | -0.001 | 0.017 |
| rs62183956 | 2 | 219046122 | T | C | NA | -0.078 | 0.013 | 4.49E-10 |  | 0.533 | -0.010 | 0.016 |
| rs62324212 | 4 | 123560939 | A | C | NA | 0.089 | 0.013 | 2.67E-12 |  | 0.011 | 0.042 | 0.016 |
| rs62408218 | 6 | 90931858 | T | C | NA | -0.082 | 0.013 | 2.40E-10 |  | 0.942 | -0.001 | 0.016 |
| rs62456432 | 7 | 10349179 | C | T | NA | -0.069 | 0.013 | 2.03E-07 |  | 0.318 | -0.017 | 0.017 |
| rs62482552 | 7 | 100522355 | A | G | NA | -0.074 | 0.013 | 1.97E-08 |  | 0.888 | -0.002 | 0.016 |
| rs6445040 | 3 | 171858523 | A | G | NA | -0.067 | 0.014 | 1.37E-06 |  | 0.474 | 0.012 | 0.016 |
| rs6469478 | 8 | 114788041 | C | G | NA | 0.061 | 0.013 | 1.43E-06 |  | 0.322 | 0.016 | 0.016 |
| rs6470877 | 8 | 132007456 | C | T | NA | 0.064 | 0.012 | 2.32E-07 |  | 0.996 | 1.00E-04 | 0.015 |
| rs6498114 | 16 | 10964118 | T | G | NA | -0.081 | 0.015 | 6.69E-08 |  | 0.373 | 0.018 | 0.020 |
| rs6584282 | 10 | 101286495 | G | A | NA | -0.152 | 0.012 | 1.19E-34 |  | 0.607 | 0.008 | 0.016 |
| rs6674040 | 1 | 20201913 | T | G | NA | -0.113 | 0.012 | 6.31E-20 |  | 0.879 | 0.002 | 0.015 |
| rs6740847 | 2 | 182308352 | G | A | NA | -0.092 | 0.013 | 1.22E-13 |  | 0.104 | 0.025 | 0.015 |
| rs6749767 | 2 | 241588985 | G | A | NA | 0.082 | 0.013 | 1.37E-10 |  | 0.369 | 0.014 | 0.016 |
| rs6770038 | 3 | 30650327 | C | T | NA | 0.087 | 0.016 | 6.67E-08 |  | 0.322 | -0.020 | 0.020 |
| rs67921626 | 16 | 50898377 | G | T | NA | 0.258 | 0.033 | 4.56E-15 |  | 0.857 | -0.006 | 0.033 |
| rs6873866 | 5 | 96247810 | C | T | NA | -0.092 | 0.013 | 6.15E-13 |  | 0.769 | -0.005 | 0.016 |
| rs6933404 | 6 | 137959235 | C | T | NA | 0.086 | 0.015 | 6.64E-09 |  | 0.304 | -0.020 | 0.020 |
| rs7171533 | 15 | 91184798 | A | G | NA | -0.110 | 0.021 | 6.79E-08 |  | 0.179 | -0.035 | 0.026 |
| rs72664358 | 1 | 57015317 | T | C | NA | 0.079 | 0.017 | 2.08E-06 |  | 0.470 | 0.015 | 0.020 |
| rs72749197 | 1 | 201056749 | C | T | NA | -0.132 | 0.024 | 3.28E-08 |  | 0.215 | 0.035 | 0.029 |
| rs72852162 | 2 | 145486323 | C | A | NA | -0.113 | 0.020 | 2.30E-08 |  | 0.668 | 0.012 | 0.028 |
| rs7319028 | 13 | 73982552 | G | A | NA | -0.105 | 0.022 | 1.12E-06 |  | 0.310 | 0.027 | 0.026 |
| rs74660460 | 9 | 93954812 | G | A | NA | -0.087 | 0.017 | 1.39E-07 |  | 0.639 | 0.010 | 0.021 |
| rs749910 | 16 | 50758849 | A | G | NA | 0.196 | 0.014 | 7.83E-46 |  | 0.439 | 0.014 | 0.018 |
| rs7512732 | 1 | 78661571 | A | G | NA | -0.185 | 0.040 | 4.57E-06 |  | 0.091 | -0.108 | 0.064 |
| rs7532161 | 1 | 67642223 | G | A | NA | 0.068 | 0.013 | 4.79E-08 |  | 0.429 | -0.012 | 0.015 |
| rs755374 | 5 | 158829294 | T | C | NA | 0.177 | 0.013 | 1.59E-39 |  | 0.026 | -0.039 | 0.018 |
| rs7608697 | 2 | 61204641 | C | A | NA | 0.140 | 0.013 | 1.67E-28 |  | 0.708 | 0.006 | 0.016 |
| rs76184771 | 19 | 18567189 | G | A | NA | 0.090 | 0.018 | 3.90E-07 |  | 0.691 | 0.009 | 0.023 |
| rs76286777 | 2 | 25195577 | C | T | NA | 0.100 | 0.015 | 4.65E-11 |  | 0.882 | -0.003 | 0.020 |
| rs7715399 | 5 | 10682230 | C | T | NA | 0.065 | 0.013 | 3.91E-07 |  | 0.807 | -0.004 | 0.016 |
| rs77272631 | 3 | 17184935 | C | G | NA | 0.229 | 0.042 | 3.72E-08 |  | 0.065 | -0.159 | 0.086 |
| rs77510039 | 4 | 106096764 | A | G | NA | -0.146 | 0.030 | 1.12E-06 |  | 0.950 | 0.002 | 0.039 |
| rs78522502 | 1 | 22723074 | G | T | NA | -0.157 | 0.027 | 7.57E-09 |  | 0.895 | -0.005 | 0.036 |
| rs78771661 | 8 | 21986850 | T | C | NA | -0.385 | 0.067 | 8.95E-09 |  | 0.569 | -0.070 | 0.123 |
| rs78827942 | 15 | 32444185 | C | T | NA | 0.095 | 0.021 | 4.11E-06 |  | 0.593 | 0.018 | 0.033 |
| rs7918084 | 10 | 94429467 | T | C | NA | 0.071 | 0.013 | 1.38E-08 |  | 0.532 | -0.010 | 0.016 |
| rs7954567 | 12 | 6491125 | A | G | NA | 0.072 | 0.015 | 2.69E-06 |  | 0.857 | -0.005 | 0.028 |
| rs8017713 | 14 | 35848845 | G | T | NA | 0.059 | 0.013 | 3.05E-06 |  | 0.441 | -0.013 | 0.017 |
| rs80262450 | 18 | 12818922 | A | G | NA | 0.158 | 0.019 | 1.04E-16 |  | 0.170 | 0.036 | 0.026 |
| rs907612 | 11 | 1874221 | T | C | NA | 0.069 | 0.013 | 2.34E-07 |  | 0.692 | 0.007 | 0.017 |
| rs916678 | 16 | 23851114 | A | C | NA | -0.085 | 0.016 | 5.22E-08 |  | 0.126 | -0.029 | 0.019 |
| rs9264942 | 6 | 31274380 | C | T | NA | 0.086 | 0.014 | 8.18E-10 |  | 0.823 | -0.005 | 0.023 |
| rs944862 | 13 | 59381163 | T | A | NA | 0.066 | 0.014 | 2.00E-06 |  | 0.517 | 0.011 | 0.018 |
| rs9594387 | 13 | 40700322 | C | G | NA | -0.068 | 0.015 | 4.09E-06 |  | 0.637 | 0.009 | 0.018 |
| rs9825587 | 3 | 149007259 | C | G | NA | -0.064 | 0.014 | 4.34E-06 |  | 0.037 | 0.036 | 0.017 |
| rs9828595 | 3 | 85893047 | G | T | NA | -0.076 | 0.015 | 8.73E-07 |  | 0.829 | -0.004 | 0.020 |

Note: SNP, single nucleotide polymorphisms; Chr, chromosome; Pos, position; EA, effect allele; OA, other allele; EAF, effect allele frequency; NA, not available; BETA, effect size; IBD, inflammatory bowel disease; SE, standard error; *P*, *P* value tests the null hypothesis of no association with exposure.

**Supplementary Table 3. Characteristics of the SNPs used as IVs in the causal association of CD with periodontitis.**

| SNP | Chr | Pos | EA | OA | EAF | Association with CD | | |  | Association with periodontitis | | |
| --- | --- | --- | --- | --- | --- | --- | --- | --- | --- | --- | --- | --- |
|  |  |  |  |  |  | BETA | SE | *P* |  | BETA | SE | *P* |
| rs10012850 | 4 | 134462621 | T | C | NA | 0.080 | 0.016 | 1.21E-06 |  | 0.013 | 0.016 | 0.425 |
| rs10114470 | 9 | 117547772 | C | T | NA | 0.169 | 0.018 | 1.76E-21 |  | -0.016 | 0.017 | 0.340 |
| rs1012636 | 6 | 20674811 | T | G | NA | 0.129 | 0.020 | 7.01E-11 |  | 0.016 | 0.019 | 0.405 |
| rs102275 | 11 | 61557803 | C | T | NA | 0.086 | 0.017 | 2.71E-07 |  | -0.014 | 0.016 | 0.401 |
| rs10423579 | 19 | 50195770 | C | T | NA | 0.087 | 0.017 | 3.79E-07 |  | -0.025 | 0.017 | 0.133 |
| rs1044690 | 6 | 44245241 | C | G | NA | -0.096 | 0.019 | 7.41E-07 |  | 0.010 | 0.018 | 0.584 |
| rs10478898 | 5 | 129134501 | T | C | NA | -0.079 | 0.017 | 2.65E-06 |  | -0.016 | 0.016 | 0.335 |
| rs10486847 | 7 | 84674997 | A | G | NA | 0.083 | 0.018 | 2.58E-06 |  | 0.016 | 0.018 | 0.388 |
| rs10798264 | 1 | 173163357 | A | G | NA | 0.097 | 0.019 | 1.60E-07 |  | 0.012 | 0.018 | 0.496 |
| rs10822050 | 10 | 64438771 | C | T | NA | 0.183 | 0.016 | 2.35E-29 |  | -0.006 | 0.016 | 0.695 |
| rs10878972 | 12 | 69777615 | C | G | NA | -0.097 | 0.021 | 3.32E-06 |  | 0.004 | 0.020 | 0.860 |
| rs10884966 | 10 | 112185596 | A | G | NA | 0.113 | 0.017 | 4.13E-11 |  | 0.002 | 0.018 | 0.931 |
| rs10916037 | 1 | 226960357 | G | A | NA | -0.077 | 0.016 | 1.71E-06 |  | -0.036 | 0.015 | 0.018 |
| rs11079258 | 17 | 54858579 | T | C | NA | 0.090 | 0.019 | 3.11E-06 |  | -0.022 | 0.020 | 0.283 |
| rs11177092 | 12 | 68585235 | G | A | NA | -0.082 | 0.016 | 3.69E-07 |  | -0.002 | 0.016 | 0.915 |
| rs11187019 | 10 | 94248310 | G | A | NA | 0.076 | 0.016 | 2.44E-06 |  | -0.007 | 0.016 | 0.671 |
| rs11214907 | 11 | 114101075 | T | C | NA | 0.141 | 0.031 | 4.96E-06 |  | 0.067 | 0.030 | 0.028 |
| rs11236797 | 11 | 76299649 | A | C | NA | 0.176 | 0.016 | 8.51E-28 |  | 0.025 | 0.016 | 0.109 |
| rs11265502 | 1 | 160837240 | G | A | NA | -0.160 | 0.029 | 4.60E-08 |  | -0.025 | 0.027 | 0.363 |
| rs112936798 | 1 | 39802381 | C | A | NA | -0.207 | 0.041 | 4.82E-07 |  | 0.043 | 0.087 | 0.624 |
| rs113756470 | 1 | 67851615 | T | C | NA | 0.326 | 0.065 | 6.08E-07 |  | -0.039 | 0.090 | 0.667 |
| rs114658781 | 3 | 121722207 | T | C | NA | 0.229 | 0.047 | 1.12E-06 |  | 0.060 | 0.054 | 0.270 |
| rs1148246 | 10 | 35496626 | T | C | NA | -0.132 | 0.017 | 2.09E-15 |  | 0.007 | 0.016 | 0.673 |
| rs115780636 | 3 | 158013408 | G | A | NA | 0.291 | 0.060 | 1.35E-06 |  | -0.071 | 0.080 | 0.376 |
| rs11683692 | 2 | 145509615 | C | T | NA | -0.214 | 0.038 | 1.75E-08 |  | 0.016 | 0.038 | 0.682 |
| rs117542833 | 18 | 2645732 | A | C | NA | 0.146 | 0.029 | 2.71E-07 |  | 0.018 | 0.030 | 0.555 |
| rs117694473 | 18 | 58856635 | G | A | NA | -0.277 | 0.061 | 4.77E-06 |  | 0.010 | 0.067 | 0.883 |
| rs12131079 | 1 | 155319668 | T | C | NA | -0.109 | 0.017 | 3.99E-10 |  | -0.017 | 0.017 | 0.329 |
| rs12421615 | 11 | 64021605 | A | G | NA | -0.083 | 0.017 | 9.90E-07 |  | 0.031 | 0.017 | 0.070 |
| rs12426498 | 12 | 40761315 | T | A | NA | -0.146 | 0.032 | 3.99E-06 |  | 0.009 | 0.027 | 0.741 |
| rs12459348 | 19 | 15543148 | T | G | NA | -0.095 | 0.019 | 5.18E-07 |  | -0.019 | 0.020 | 0.334 |
| rs1250573 | 10 | 81042475 | A | G | NA | -0.152 | 0.018 | 1.92E-17 |  | -0.016 | 0.018 | 0.353 |
| rs12936409 | 17 | 38043649 | T | C | NA | 0.143 | 0.016 | 4.31E-19 |  | -0.004 | 0.016 | 0.801 |
| rs1297264 | 21 | 16816017 | G | A | NA | -0.177 | 0.016 | 1.59E-27 |  | 6.00E-04 | 0.016 | 0.970 |
| rs13019195 | 2 | 141161818 | A | G | NA | 0.120 | 0.026 | 2.77E-06 |  | -0.001 | 0.026 | 0.959 |
| rs1321859 | 6 | 91011673 | T | C | NA | -0.105 | 0.017 | 1.18E-09 |  | -0.002 | 0.017 | 0.912 |
| rs1373904 | 13 | 44475398 | G | A | NA | 0.141 | 0.019 | 9.11E-14 |  | 0.022 | 0.018 | 0.214 |
| rs139350478 | 6 | 63893901 | A | T | NA | -0.337 | 0.071 | 2.25E-06 |  | -0.015 | 0.084 | 0.858 |
| rs140313246 | 17 | 7007622 | A | G | NA | -0.179 | 0.035 | 3.43E-07 |  | 0.016 | 0.030 | 0.585 |
| rs144309607 | 19 | 10492274 | T | C | NA | -0.371 | 0.047 | 2.69E-15 |  | -0.043 | 0.054 | 0.428 |
| rs144341967 | 7 | 98748135 | G | T | NA | 0.178 | 0.038 | 2.92E-06 |  | -0.015 | 0.039 | 0.706 |
| rs149174226 | 14 | 58322375 | T | A | NA | 0.399 | 0.083 | 1.36E-06 |  | -0.047 | 0.109 | 0.667 |
| rs150649461 | 1 | 92925654 | C | G | NA | -0.398 | 0.082 | 1.27E-06 |  | 0.166 | 0.095 | 0.081 |
| rs1583792 | 2 | 198900288 | T | C | NA | -0.088 | 0.016 | 3.26E-08 |  | 0.002 | 0.015 | 0.876 |
| rs16861305 | 4 | 48466781 | A | G | NA | -0.076 | 0.016 | 2.35E-06 |  | 0.014 | 0.016 | 0.360 |
| rs17445836 | 16 | 86017663 | A | G | NA | -0.107 | 0.021 | 1.84E-07 |  | -0.036 | 0.023 | 0.113 |
| rs17643535 | 8 | 18412349 | C | G | NA | 0.360 | 0.074 | 1.18E-06 |  | -0.019 | 0.042 | 0.655 |
| rs17652674 | 15 | 38900312 | A | C | NA | 0.096 | 0.020 | 1.25E-06 |  | -0.023 | 0.020 | 0.252 |
| rs17885629 | 17 | 40525098 | G | A | NA | -0.114 | 0.016 | 2.05E-12 |  | 0.020 | 0.016 | 0.212 |
| rs1788649 | 18 | 77555233 | C | T | NA | -0.078 | 0.017 | 2.55E-06 |  | 0.020 | 0.016 | 0.216 |
| rs181826 | 5 | 141526057 | A | C | NA | 0.116 | 0.017 | 3.24E-12 |  | 0.013 | 0.016 | 0.425 |
| rs1887428 | 9 | 4984530 | C | G | NA | -0.166 | 0.017 | 8.54E-23 |  | 0.003 | 0.016 | 0.844 |
| rs194746 | 14 | 69282887 | T | C | NA | 0.098 | 0.016 | 1.24E-09 |  | 0.007 | 0.016 | 0.661 |
| rs2021511 | 16 | 11344903 | T | C | NA | -0.108 | 0.018 | 2.63E-09 |  | -0.002 | 0.018 | 0.903 |
| rs2063113 | 1 | 25303762 | G | T | NA | -0.087 | 0.017 | 2.79E-07 |  | 0.017 | 0.017 | 0.303 |
| rs2076756 | 16 | 50756881 | G | A | NA | 0.385 | 0.017 | ######## |  | 0.014 | 0.018 | 0.439 |
| rs210609 | 6 | 117833361 | A | T | NA | 0.084 | 0.017 | 1.48E-06 |  | -0.007 | 0.016 | 0.683 |
| rs2110735 | 2 | 103050925 | G | A | NA | -0.137 | 0.019 | 1.20E-13 |  | -0.015 | 0.018 | 0.398 |
| rs212388 | 6 | 159490436 | T | C | NA | -0.107 | 0.017 | 9.52E-11 |  | 0.001 | 0.016 | 0.928 |
| rs2143178 | 22 | 39660829 | C | T | NA | -0.209 | 0.022 | 6.84E-21 |  | -0.007 | 0.021 | 0.740 |
| rs2188962 | 5 | 131770805 | T | C | NA | 0.200 | 0.016 | 5.59E-36 |  | 1.00E-04 | 0.016 | 0.994 |
| rs2257745 | 10 | 19718264 | T | C | NA | 0.091 | 0.020 | 3.13E-06 |  | 0.006 | 0.021 | 0.781 |
| rs2284553 | 21 | 34776695 | G | A | NA | 0.128 | 0.017 | 1.14E-14 |  | -0.016 | 0.016 | 0.305 |
| rs237949 | 16 | 1041856 | C | G | NA | 0.248 | 0.053 | 3.13E-06 |  | 0.012 | 0.052 | 0.818 |
| rs2390314 | 7 | 20455978 | T | A | NA | -0.166 | 0.031 | 8.02E-08 |  | -0.019 | 0.030 | 0.515 |
| rs243541 | 7 | 148403243 | T | A | NA | -0.087 | 0.017 | 1.54E-07 |  | -0.018 | 0.016 | 0.256 |
| rs2476601 | 1 | 114377568 | G | A | NA | 0.231 | 0.029 | 6.44E-16 |  | -0.011 | 0.028 | 0.699 |
| rs2494271 | 1 | 197347920 | T | C | NA | 0.112 | 0.017 | 5.17E-11 |  | 0.020 | 0.017 | 0.247 |
| rs2526355 | 17 | 57953233 | C | G | NA | 0.078 | 0.016 | 9.06E-07 |  | 0.011 | 0.016 | 0.472 |
| rs259975 | 20 | 57809838 | C | T | NA | -0.076 | 0.016 | 3.37E-06 |  | 0.004 | 0.016 | 0.817 |
| rs2675670 | 10 | 75655628 | C | G | NA | 0.107 | 0.016 | 2.89E-11 |  | -0.011 | 0.016 | 0.483 |
| rs2838517 | 21 | 45613825 | C | T | NA | -0.146 | 0.016 | 2.03E-19 |  | -0.003 | 0.016 | 0.845 |
| rs28722705 | 5 | 55453942 | T | A | NA | -0.125 | 0.024 | 1.15E-07 |  | 0.037 | 0.023 | 0.111 |
| rs2879179 | 2 | 28606778 | T | C | NA | 0.109 | 0.016 | 2.82E-11 |  | 0.009 | 0.016 | 0.585 |
| rs28999107 | 12 | 6493100 | T | G | NA | 0.108 | 0.018 | 1.06E-09 |  | -9.00E-04 | 0.022 | 0.966 |
| rs2948542 | 17 | 25856486 | G | A | NA | 0.102 | 0.016 | 5.15E-10 |  | 0.011 | 0.016 | 0.487 |
| rs3122605 | 1 | 206955041 | A | G | NA | -0.175 | 0.023 | 1.24E-14 |  | 0.020 | 0.023 | 0.393 |
| rs34004493 | 2 | 231154012 | G | A | NA | 0.126 | 0.018 | 2.00E-12 |  | -0.017 | 0.019 | 0.373 |
| rs34014701 | 5 | 72554768 | G | A | NA | -0.078 | 0.016 | 1.38E-06 |  | -0.013 | 0.016 | 0.419 |
| rs341295 | 5 | 111848890 | T | C | NA | 0.080 | 0.016 | 6.59E-07 |  | -5.00E-04 | 0.016 | 0.973 |
| rs34217989 | 5 | 158564207 | C | T | NA | -0.090 | 0.018 | 7.48E-07 |  | -0.034 | 0.018 | 0.062 |
| rs34471628 | 5 | 172196752 | G | A | NA | -0.229 | 0.047 | 1.44E-06 |  | -0.043 | 0.044 | 0.326 |
| rs34517439 | 1 | 78450517 | A | C | NA | -0.125 | 0.027 | 4.65E-06 |  | -0.018 | 0.030 | 0.550 |
| rs34635748 | 12 | 40824663 | T | C | NA | 0.479 | 0.050 | 1.95E-21 |  | 0.053 | 0.039 | 0.170 |
| rs348869 | 3 | 100987978 | G | A | NA | -0.081 | 0.016 | 4.33E-07 |  | -0.007 | 0.016 | 0.633 |
| rs35171809 | 6 | 167432766 | G | A | NA | 0.157 | 0.016 | 9.07E-23 |  | 0.014 | 0.016 | 0.383 |
| rs36032938 | 8 | 90799961 | A | G | NA | -0.155 | 0.030 | 1.77E-07 |  | -0.018 | 0.028 | 0.517 |
| rs3761158 | 20 | 44634912 | A | G | NA | -0.110 | 0.017 | 2.65E-11 |  | -0.023 | 0.016 | 0.141 |
| rs3816234 | 2 | 234185999 | A | G | NA | 0.270 | 0.016 | 1.51E-62 |  | 0.002 | 0.016 | 0.921 |
| rs3850378 | 14 | 88417517 | C | T | NA | 0.199 | 0.027 | 8.31E-14 |  | 0.023 | 0.025 | 0.371 |
| rs4072601 | 17 | 70641327 | A | G | NA | -0.106 | 0.023 | 3.58E-06 |  | -0.005 | 0.021 | 0.804 |
| rs4077515 | 9 | 139266496 | T | C | NA | 0.185 | 0.016 | 3.14E-30 |  | 0.003 | 0.016 | 0.851 |
| rs42861 | 16 | 28494421 | G | A | NA | 0.124 | 0.017 | 8.87E-14 |  | 0.026 | 0.019 | 0.180 |
| rs4317904 | 10 | 50294670 | A | G | NA | 0.077 | 0.017 | 3.63E-06 |  | -0.003 | 0.016 | 0.863 |
| rs4343432 | 2 | 25161236 | G | A | NA | 0.112 | 0.016 | 3.50E-12 |  | 0.009 | 0.016 | 0.564 |
| rs4788456 | 16 | 72047286 | A | G | NA | 0.081 | 0.017 | 2.22E-06 |  | -0.003 | 0.016 | 0.879 |
| rs4807570 | 19 | 1123652 | A | G | NA | 0.181 | 0.019 | 6.03E-21 |  | -0.005 | 0.018 | 0.805 |
| rs4833071 | 4 | 38582859 | C | T | NA | 0.094 | 0.017 | 7.00E-08 |  | -0.022 | 0.017 | 0.196 |
| rs492602 | 19 | 49206417 | G | A | NA | 0.108 | 0.016 | 2.33E-11 |  | -0.002 | 0.016 | 0.920 |
| rs543379 | 9 | 265469 | G | T | NA | -0.081 | 0.016 | 4.96E-07 |  | 0.009 | 0.016 | 0.585 |
| rs55946629 | 2 | 43851246 | A | C | NA | 0.176 | 0.023 | 2.85E-14 |  | 0.002 | 0.023 | 0.946 |
| rs56116661 | 3 | 188401160 | T | C | NA | -0.131 | 0.021 | 5.67E-10 |  | -0.015 | 0.020 | 0.468 |
| rs5743704 | 4 | 154625951 | A | C | NA | 0.192 | 0.042 | 4.92E-06 |  | -0.010 | 0.047 | 0.835 |
| rs5754100 | 22 | 21916166 | C | T | NA | 0.169 | 0.021 | 3.02E-16 |  | -0.022 | 0.022 | 0.308 |
| rs59926756 | 16 | 82879772 | A | G | NA | 0.106 | 0.018 | 1.74E-09 |  | -0.008 | 0.017 | 0.650 |
| rs6062496 | 20 | 62329099 | A | G | NA | 0.122 | 0.017 | 2.62E-13 |  | 0.020 | 0.017 | 0.238 |
| rs61629455 | 10 | 23647845 | G | A | NA | 0.167 | 0.036 | 4.06E-06 |  | 0.056 | 0.030 | 0.061 |
| rs61839660 | 10 | 6094697 | T | C | NA | 0.147 | 0.026 | 1.98E-08 |  | -0.019 | 0.028 | 0.490 |
| rs62126620 | 19 | 33753200 | A | G | NA | 0.144 | 0.020 | 8.61E-13 |  | -0.017 | 0.020 | 0.392 |
| rs62202889 | 20 | 14544626 | A | G | NA | -0.099 | 0.019 | 7.69E-08 |  | 0.014 | 0.019 | 0.456 |
| rs62324212 | 4 | 123560939 | A | C | NA | 0.106 | 0.016 | 8.02E-11 |  | 0.042 | 0.016 | 0.011 |
| rs6451494 | 5 | 40411291 | C | T | NA | 0.261 | 0.017 | 8.26E-56 |  | 0.034 | 0.016 | 0.032 |
| rs6550006 | 3 | 30652788 | A | G | NA | 0.105 | 0.021 | 5.18E-07 |  | -0.023 | 0.021 | 0.265 |
| rs6584282 | 10 | 101286495 | G | A | NA | -0.166 | 0.016 | 3.44E-25 |  | 0.008 | 0.016 | 0.607 |
| rs6704109 | 1 | 172857050 | T | C | NA | 0.175 | 0.018 | 5.10E-22 |  | 0.014 | 0.018 | 0.430 |
| rs6734942 | 2 | 111910694 | T | C | NA | -0.082 | 0.017 | 1.93E-06 |  | 0.032 | 0.017 | 0.052 |
| rs6740847 | 2 | 182308352 | G | A | NA | -0.104 | 0.016 | 9.72E-11 |  | 0.025 | 0.015 | 0.104 |
| rs67921626 | 16 | 50898377 | G | T | NA | 0.522 | 0.039 | 1.06E-40 |  | -0.006 | 0.033 | 0.857 |
| rs6808936 | 3 | 141109321 | G | A | NA | 0.090 | 0.016 | 1.93E-08 |  | 0.031 | 0.016 | 0.044 |
| rs6873866 | 5 | 96247810 | C | T | NA | -0.131 | 0.016 | 1.35E-15 |  | -0.005 | 0.016 | 0.769 |
| rs697693 | 1 | 7886424 | A | G | NA | 0.107 | 0.020 | 7.45E-08 |  | 0.029 | 0.020 | 0.138 |
| rs7149392 | 14 | 35856876 | G | C | NA | -0.087 | 0.017 | 1.93E-07 |  | 0.014 | 0.017 | 0.420 |
| rs7157631 | 14 | 103873325 | C | T | NA | 0.088 | 0.017 | 1.95E-07 |  | 0.021 | 0.017 | 0.208 |
| rs7176192 | 15 | 26016067 | C | T | NA | 0.087 | 0.019 | 3.63E-06 |  | 0.015 | 0.019 | 0.423 |
| rs7195228 | 16 | 50353529 | G | C | NA | -0.133 | 0.021 | 2.09E-10 |  | -0.004 | 0.019 | 0.829 |
| rs7240004 | 18 | 46395022 | G | A | NA | -0.080 | 0.017 | 3.10E-06 |  | -0.018 | 0.016 | 0.245 |
| rs72743461 | 15 | 67441750 | A | C | NA | 0.168 | 0.019 | 2.26E-19 |  | 0.020 | 0.019 | 0.281 |
| rs73120731 | 7 | 37457483 | G | A | NA | 0.112 | 0.024 | 3.56E-06 |  | 0.004 | 0.025 | 0.868 |
| rs73243877 | 4 | 26047616 | G | A | NA | 0.116 | 0.021 | 4.12E-08 |  | -2.00E-04 | 0.022 | 0.993 |
| rs73516754 | 6 | 106459738 | C | A | NA | 0.142 | 0.017 | 4.04E-17 |  | -0.003 | 0.017 | 0.882 |
| rs74755331 | 2 | 40159310 | T | G | NA | 0.157 | 0.033 | 1.88E-06 |  | -0.063 | 0.035 | 0.077 |
| rs7517847 | 1 | 67681669 | G | T | NA | -0.345 | 0.017 | 5.84E-97 |  | 0.015 | 0.016 | 0.320 |
| rs75339988 | 10 | 126544811 | T | A | NA | -0.126 | 0.026 | 1.23E-06 |  | -0.023 | 0.024 | 0.337 |
| rs755374 | 5 | 158829294 | T | C | NA | 0.197 | 0.017 | 1.38E-29 |  | -0.039 | 0.018 | 0.026 |
| rs7576339 | 2 | 242490882 | A | G | NA | -0.093 | 0.020 | 3.58E-06 |  | -0.020 | 0.022 | 0.357 |
| rs760517 | 22 | 37258986 | T | C | NA | 0.096 | 0.018 | 7.18E-08 |  | 0.006 | 0.017 | 0.706 |
| rs7608697 | 2 | 61204641 | C | A | NA | 0.123 | 0.016 | 4.03E-14 |  | 0.006 | 0.016 | 0.708 |
| rs7715399 | 5 | 10682230 | C | T | NA | 0.088 | 0.017 | 1.34E-07 |  | -0.004 | 0.016 | 0.807 |
| rs77566919 | 12 | 113163656 | A | G | NA | -0.109 | 0.019 | 4.13E-09 |  | -0.017 | 0.021 | 0.411 |
| rs77938224 | 2 | 216848461 | A | G | NA | -0.228 | 0.050 | 4.52E-06 |  | 0.047 | 0.082 | 0.569 |
| rs7814134 | 8 | 129497003 | A | G | NA | -0.132 | 0.025 | 1.90E-07 |  | -3.00E-04 | 0.023 | 0.991 |
| rs79046358 | 7 | 117248542 | C | T | NA | -0.206 | 0.042 | 1.05E-06 |  | -0.025 | 0.045 | 0.575 |
| rs79325872 | 5 | 40508737 | C | T | NA | -0.159 | 0.024 | 7.55E-11 |  | 0.018 | 0.024 | 0.456 |
| rs79832570 | 8 | 145097720 | C | T | NA | 0.223 | 0.034 | 8.90E-11 |  | 0.067 | 0.059 | 0.255 |
| rs80262450 | 18 | 12818922 | A | G | NA | 0.227 | 0.024 | 1.34E-20 |  | 0.036 | 0.026 | 0.170 |
| rs869310 | 15 | 77830306 | G | T | NA | -0.079 | 0.017 | 3.90E-06 |  | -0.012 | 0.016 | 0.469 |
| rs882080 | 10 | 30804597 | A | T | NA | -0.127 | 0.019 | 1.24E-11 |  | -0.011 | 0.018 | 0.544 |
| rs891369 | 3 | 53136551 | A | G | NA | 0.084 | 0.018 | 1.73E-06 |  | -0.030 | 0.018 | 0.091 |
| rs9264942 | 6 | 31274380 | C | T | NA | 0.154 | 0.018 | 5.31E-18 |  | -0.005 | 0.023 | 0.823 |
| rs9466056 | 6 | 21384613 | G | A | NA | -0.091 | 0.016 | 2.57E-08 |  | -0.019 | 0.016 | 0.223 |
| rs9482770 | 6 | 127443092 | C | T | NA | 0.099 | 0.016 | 1.01E-09 |  | -0.005 | 0.016 | 0.738 |
| rs9533108 | 13 | 43024710 | T | C | NA | -0.085 | 0.016 | 1.16E-07 |  | -0.024 | 0.016 | 0.130 |
| rs9557201 | 13 | 99989538 | G | A | NA | -0.082 | 0.018 | 4.17E-06 |  | -0.003 | 0.017 | 0.875 |
| rs9656588 | 7 | 50306780 | C | T | NA | 0.118 | 0.017 | 8.73E-12 |  | -1.00E-04 | 0.017 | 0.997 |
| rs977004 | 1 | 185344764 | G | A | NA | -0.077 | 0.017 | 3.90E-06 |  | -0.020 | 0.016 | 0.216 |
| rs9836291 | 3 | 49697459 | A | G | NA | 0.172 | 0.017 | 3.77E-24 |  | 0.004 | 0.017 | 0.841 |
| rs9908756 | 17 | 81043039 | G | A | NA | -0.091 | 0.018 | 3.96E-07 |  | -0.047 | 0.019 | 0.013 |

Note: SNP, single nucleotide polymorphisms; Chr, chromosome; Pos, position; EA, effect allele; OA, other allele; EAF, effect allele frequency; NA, not available; BETA, effect size; CD, Crohn’s disease; SE, standard error; *P*, *P* value tests the null hypothesis of no association with exposure.

**Supplementary Table 4. Characteristics of the SNPs used as IVs in the causal association of UC with periodontitis.**

| SNP | Chr | Pos | EA | OA | EAF | Association with UC | | |  | Association with periodontitis | | |
| --- | --- | --- | --- | --- | --- | --- | --- | --- | --- | --- | --- | --- |
|  |  |  |  |  |  | BETA | SE | *P* |  | BETA | SE | *P* |
| rs10272963 | 7 | 107486902 | T | C | NA | -0.151 | 0.016 | 4.11E-21 |  | 0.007 | 0.016 | 0.675 |
| rs10408351 | 19 | 33754044 | A | G | NA | 0.155 | 0.020 | 2.92E-14 |  | -0.008 | 0.021 | 0.708 |
| rs10737481 | 1 | 20171514 | G | T | NA | 0.217 | 0.016 | 2.56E-42 |  | -0.003 | 0.015 | 0.832 |
| rs10741849 | 11 | 20643212 | A | G | NA | 0.075 | 0.016 | 1.98E-06 |  | -0.005 | 0.016 | 0.765 |
| rs10761659 | 10 | 64445564 | G | A | NA | 0.128 | 0.016 | 1.33E-15 |  | -0.017 | 0.016 | 0.278 |
| rs10799374 | 1 | 227102033 | T | C | NA | 0.078 | 0.016 | 1.02E-06 |  | 0.005 | 0.016 | 0.771 |
| rs10817678 | 9 | 117579457 | A | G | NA | 0.133 | 0.017 | 4.42E-15 |  | -0.017 | 0.017 | 0.320 |
| rs10917545 | 1 | 20128177 | A | G | NA | -0.223 | 0.025 | 7.03E-19 |  | 0.008 | 0.026 | 0.751 |
| rs111407861 | 6 | 93517951 | T | C | NA | 0.152 | 0.030 | 5.94E-07 |  | 0.035 | 0.029 | 0.236 |
| rs111672122 | 7 | 76490055 | G | T | NA | -0.084 | 0.018 | 4.65E-06 |  | -0.031 | 0.026 | 0.236 |
| rs11168249 | 12 | 48208368 | C | T | NA | 0.084 | 0.016 | 3.18E-07 |  | -0.010 | 0.016 | 0.517 |
| rs11209026 | 1 | 67705958 | A | G | NA | -0.483 | 0.036 | 1.99E-41 |  | 0.040 | 0.032 | 0.211 |
| rs112124254 | 1 | 46822147 | A | G | NA | -0.108 | 0.021 | 2.70E-07 |  | 0.024 | 0.024 | 0.319 |
| rs1131095 | 3 | 49714225 | C | T | NA | 0.159 | 0.017 | 2.18E-21 |  | 0.002 | 0.017 | 0.928 |
| rs113986290 | 6 | 19781009 | T | C | NA | -0.307 | 0.053 | 7.59E-09 |  | -0.027 | 0.051 | 0.600 |
| rs115803787 | 17 | 6797932 | T | A | NA | 0.257 | 0.051 | 3.95E-07 |  | -0.081 | 0.057 | 0.159 |
| rs11645239 | 16 | 23847062 | G | C | NA | -0.117 | 0.020 | 4.14E-09 |  | -0.026 | 0.019 | 0.175 |
| rs116935315 | 7 | 148455040 | T | C | NA | 0.320 | 0.069 | 3.10E-06 |  | 0.109 | 0.087 | 0.215 |
| rs117136834 | 13 | 40826366 | A | G | NA | -0.198 | 0.037 | 8.13E-08 |  | -0.048 | 0.039 | 0.215 |
| rs118014971 | 9 | 71339178 | G | T | NA | 0.467 | 0.096 | 1.15E-06 |  | 0.230 | 0.157 | 0.143 |
| rs118176029 | 12 | 21336573 | C | T | NA | -0.271 | 0.057 | 2.09E-06 |  | 0.113 | 0.092 | 0.220 |
| rs11981405 | 7 | 73162437 | G | A | NA | 0.094 | 0.018 | 1.77E-07 |  | 0.024 | 0.017 | 0.165 |
| rs12283414 | 11 | 58176920 | T | C | NA | -0.098 | 0.020 | 6.04E-07 |  | 0.036 | 0.019 | 0.057 |
| rs12435718 | 14 | 69279879 | G | A | NA | -0.081 | 0.017 | 2.81E-06 |  | -0.013 | 0.017 | 0.450 |
| rs12645566 | 4 | 156747798 | C | A | NA | 0.131 | 0.026 | 6.41E-07 |  | -0.003 | 0.027 | 0.926 |
| rs1267500 | 6 | 14715825 | T | C | NA | 0.103 | 0.021 | 4.76E-07 |  | -0.023 | 0.019 | 0.223 |
| rs12825700 | 12 | 68492980 | A | G | NA | 0.189 | 0.016 | 7.33E-32 |  | 0.003 | 0.017 | 0.882 |
| rs12936409 | 17 | 38043649 | T | C | NA | 0.137 | 0.016 | 5.62E-18 |  | -0.004 | 0.016 | 0.801 |
| rs12985060 | 19 | 47145658 | C | T | NA | -0.093 | 0.019 | 1.07E-06 |  | -0.033 | 0.019 | 0.078 |
| rs13022757 | 2 | 102613546 | C | G | NA | -0.083 | 0.016 | 1.40E-07 |  | 0.015 | 0.016 | 0.346 |
| rs1319951 | 18 | 46404813 | G | C | NA | -0.091 | 0.019 | 1.27E-06 |  | -0.026 | 0.019 | 0.155 |
| rs13200059 | 6 | 111943234 | A | G | NA | 0.294 | 0.044 | 1.48E-11 |  | 0.030 | 0.047 | 0.522 |
| rs13406089 | 2 | 163326297 | A | G | NA | 0.089 | 0.018 | 6.52E-07 |  | -0.041 | 0.019 | 0.033 |
| rs1359105 | 9 | 100870501 | C | G | NA | 0.103 | 0.020 | 2.73E-07 |  | -0.018 | 0.020 | 0.359 |
| rs1359946 | 13 | 27536972 | A | G | NA | 0.157 | 0.020 | 6.58E-15 |  | -0.009 | 0.020 | 0.670 |
| rs137845 | 22 | 50439430 | G | A | NA | 0.101 | 0.016 | 1.50E-10 |  | -0.007 | 0.016 | 0.636 |
| rs139839221 | 4 | 84230033 | T | C | NA | 0.331 | 0.064 | 2.22E-07 |  | -0.205 | 0.121 | 0.091 |
| rs140152928 | 2 | 132143532 | A | T | NA | -0.307 | 0.066 | 3.17E-06 |  | 0.290 | 0.164 | 0.077 |
| rs140900359 | 6 | 69009744 | C | T | NA | 0.378 | 0.072 | 1.49E-07 |  | 0.064 | 0.116 | 0.580 |
| rs149020882 | 16 | 50710388 | T | C | NA | 0.338 | 0.066 | 2.97E-07 |  | 0.032 | 0.098 | 0.745 |
| rs1492479 | 3 | 111860546 | C | A | NA | -0.093 | 0.020 | 3.93E-06 |  | 0.015 | 0.021 | 0.469 |
| rs149960148 | 20 | 32867420 | A | G | NA | 0.316 | 0.068 | 3.07E-06 |  | 0.075 | 0.089 | 0.401 |
| rs16940186 | 16 | 86009740 | C | T | NA | 0.136 | 0.021 | 2.18E-10 |  | 0.027 | 0.024 | 0.269 |
| rs17038630 | 4 | 109056791 | T | G | NA | 0.139 | 0.029 | 2.26E-06 |  | -0.026 | 0.029 | 0.355 |
| rs1736161 | 21 | 16833222 | A | G | NA | -0.123 | 0.016 | 2.22E-14 |  | 0.001 | 0.016 | 0.950 |
| rs17700081 | 4 | 26198829 | A | G | NA | 0.099 | 0.020 | 6.22E-07 |  | -0.021 | 0.020 | 0.284 |
| rs17715902 | 5 | 134451465 | A | G | NA | 0.097 | 0.017 | 4.62E-09 |  | 0.017 | 0.016 | 0.299 |
| rs17776393 | 14 | 50574019 | A | G | NA | -0.342 | 0.074 | 4.23E-06 |  | 0.028 | 0.090 | 0.756 |
| rs1786707 | 11 | 96031872 | A | T | NA | 0.087 | 0.018 | 6.08E-07 |  | -0.004 | 0.018 | 0.810 |
| rs1811711 | 2 | 228670476 | G | C | NA | -0.130 | 0.022 | 6.09E-09 |  | 0.023 | 0.025 | 0.355 |
| rs185627103 | 3 | 141595378 | G | T | NA | 0.105 | 0.022 | 2.70E-06 |  | -0.014 | 0.023 | 0.558 |
| rs1887428 | 9 | 4984530 | C | G | NA | -0.167 | 0.017 | 9.65E-24 |  | 0.003 | 0.016 | 0.844 |
| rs1990134 | 7 | 41974917 | T | C | NA | 0.083 | 0.017 | 8.21E-07 |  | 0.016 | 0.017 | 0.342 |
| rs1991866 | 8 | 130624105 | C | G | NA | -0.076 | 0.016 | 2.21E-06 |  | 0.006 | 0.016 | 0.717 |
| rs1995301 | 1 | 70991829 | G | A | NA | -0.081 | 0.017 | 1.36E-06 |  | 0.013 | 0.017 | 0.454 |
| rs2045241 | 11 | 114428783 | A | G | NA | -0.106 | 0.017 | 2.83E-10 |  | 0.007 | 0.016 | 0.650 |
| rs2201103 | 3 | 63781484 | C | T | NA | -0.095 | 0.021 | 3.99E-06 |  | 0.011 | 0.021 | 0.592 |
| rs2212434 | 11 | 76281593 | T | C | NA | 0.125 | 0.016 | 2.80E-15 |  | 0.024 | 0.015 | 0.119 |
| rs2285459 | 16 | 30495412 | C | T | NA | -0.079 | 0.016 | 9.43E-07 |  | -0.006 | 0.016 | 0.719 |
| rs2813773 | 1 | 110364821 | A | G | NA | 0.079 | 0.017 | 2.88E-06 |  | 0.005 | 0.016 | 0.764 |
| rs2816971 | 1 | 200085376 | A | G | NA | 0.168 | 0.025 | 1.54E-11 |  | 0.013 | 0.024 | 0.605 |
| rs2836881 | 21 | 40466299 | T | G | NA | -0.222 | 0.019 | 1.11E-32 |  | -0.002 | 0.017 | 0.910 |
| rs28516832 | 15 | 41367036 | A | G | NA | -0.098 | 0.019 | 1.10E-07 |  | -0.028 | 0.019 | 0.146 |
| rs3024493 | 1 | 206943968 | A | C | NA | 0.210 | 0.021 | 7.46E-24 |  | -0.028 | 0.022 | 0.212 |
| rs34119476 | 2 | 199584179 | G | A | NA | 0.100 | 0.016 | 3.64E-10 |  | -0.006 | 0.016 | 0.714 |
| rs34266615 | 5 | 149600519 | A | G | NA | 0.084 | 0.016 | 1.90E-07 |  | 5.00E-04 | 0.016 | 0.974 |
| rs3804032 | 21 | 45629691 | G | A | NA | -0.105 | 0.016 | 6.88E-11 |  | 0.005 | 0.016 | 0.736 |
| rs3812565 | 9 | 139272502 | C | T | NA | 0.134 | 0.016 | 6.50E-17 |  | -0.006 | 0.016 | 0.723 |
| rs394408 | 4 | 74849352 | C | T | NA | 0.130 | 0.026 | 3.75E-07 |  | -0.027 | 0.025 | 0.278 |
| rs41162 | 22 | 30408710 | T | C | NA | 0.096 | 0.018 | 7.06E-08 |  | 0.002 | 0.017 | 0.917 |
| rs4236540 | 7 | 98769025 | T | G | NA | -0.087 | 0.018 | 8.07E-07 |  | 0.007 | 0.017 | 0.680 |
| rs4462528 | 14 | 88426297 | C | T | NA | 0.127 | 0.026 | 1.11E-06 |  | 0.019 | 0.025 | 0.456 |
| rs45605540 | 4 | 123141054 | C | T | NA | 0.093 | 0.018 | 1.02E-07 |  | 0.036 | 0.018 | 0.048 |
| rs4676408 | 2 | 241574401 | A | G | NA | 0.143 | 0.017 | 1.19E-17 |  | 0.015 | 0.016 | 0.351 |
| rs4722672 | 7 | 27231762 | T | C | NA | -0.108 | 0.021 | 1.26E-07 |  | -0.003 | 0.019 | 0.895 |
| rs4728142 | 7 | 128573967 | A | G | NA | 0.100 | 0.016 | 3.23E-10 |  | 0.024 | 0.016 | 0.124 |
| rs4915452 | 1 | 200677362 | A | G | NA | -0.085 | 0.016 | 1.32E-07 |  | -0.011 | 0.016 | 0.510 |
| rs4962658 | 10 | 126265376 | A | C | NA | -0.126 | 0.025 | 2.52E-07 |  | -0.006 | 0.023 | 0.791 |
| rs56062135 | 15 | 67455630 | T | C | NA | 0.108 | 0.018 | 4.66E-09 |  | 0.019 | 0.019 | 0.317 |
| rs57266556 | 6 | 43798981 | T | C | NA | 0.103 | 0.022 | 4.56E-06 |  | 0.012 | 0.022 | 0.570 |
| rs6017342 | 20 | 43065028 | C | A | NA | 0.194 | 0.017 | 3.95E-30 |  | -0.016 | 0.019 | 0.403 |
| rs6062496 | 20 | 62329099 | A | G | NA | 0.136 | 0.016 | 8.97E-17 |  | 0.020 | 0.017 | 0.238 |
| rs62180181 | 2 | 199852010 | T | C | NA | 0.123 | 0.017 | 8.08E-13 |  | -0.030 | 0.018 | 0.096 |
| rs626893 | 4 | 38114374 | T | C | NA | 0.082 | 0.017 | 7.50E-07 |  | -0.011 | 0.017 | 0.502 |
| rs6503695 | 17 | 40499533 | C | T | NA | -0.108 | 0.017 | 1.04E-10 |  | 0.008 | 0.017 | 0.649 |
| rs6658353 | 1 | 161469054 | C | G | NA | -0.157 | 0.016 | 1.17E-22 |  | -0.011 | 0.015 | 0.489 |
| rs66988573 | 6 | 170418643 | G | C | NA | 0.107 | 0.023 | 1.77E-06 |  | 0.013 | 0.024 | 0.580 |
| rs67111717 | 5 | 176790162 | G | A | NA | 0.094 | 0.017 | 3.27E-08 |  | 0.001 | 0.017 | 0.942 |
| rs6731125 | 2 | 182308836 | C | T | NA | -0.077 | 0.016 | 1.31E-06 |  | 0.026 | 0.016 | 0.097 |
| rs6737563 | 2 | 218945674 | C | T | NA | 0.082 | 0.016 | 4.17E-07 |  | 0.001 | 0.017 | 0.952 |
| rs6808275 | 3 | 188491558 | G | A | NA | 0.086 | 0.018 | 7.89E-07 |  | 0.010 | 0.017 | 0.548 |
| rs6889364 | 5 | 40347469 | A | G | NA | 0.132 | 0.023 | 7.87E-09 |  | 0.050 | 0.023 | 0.032 |
| rs6933404 | 6 | 137959235 | C | T | NA | 0.149 | 0.019 | 2.69E-15 |  | -0.020 | 0.020 | 0.304 |
| rs7093419 | 10 | 8509152 | C | T | NA | 0.160 | 0.029 | 5.40E-08 |  | -0.014 | 0.027 | 0.617 |
| rs7171533 | 15 | 91184798 | A | G | NA | -0.127 | 0.026 | 1.18E-06 |  | -0.035 | 0.026 | 0.179 |
| rs7203363 | 16 | 68587692 | A | T | NA | 0.107 | 0.019 | 1.41E-08 |  | -0.012 | 0.018 | 0.486 |
| rs72704802 | 5 | 554211 | T | C | NA | -0.122 | 0.021 | 2.89E-09 |  | 0.013 | 0.022 | 0.568 |
| rs72787323 | 10 | 53983697 | C | T | NA | 0.165 | 0.036 | 3.30E-06 |  | 0.022 | 0.037 | 0.559 |
| rs7544646 | 1 | 2496649 | G | C | NA | -0.117 | 0.016 | 2.53E-13 |  | -0.026 | 0.015 | 0.092 |
| rs755374 | 5 | 158829294 | T | C | NA | 0.171 | 0.017 | 9.73E-24 |  | -0.039 | 0.018 | 0.026 |
| rs7605725 | 2 | 43641627 | G | A | NA | -0.087 | 0.017 | 2.26E-07 |  | -0.017 | 0.017 | 0.309 |
| rs7608697 | 2 | 61204641 | C | A | NA | 0.160 | 0.016 | 3.03E-23 |  | 0.006 | 0.016 | 0.708 |
| rs7694145 | 4 | 177285894 | G | A | NA | 0.098 | 0.021 | 3.07E-06 |  | 0.017 | 0.021 | 0.403 |
| rs77272631 | 3 | 17184935 | C | G | NA | 0.255 | 0.054 | 2.18E-06 |  | -0.159 | 0.086 | 0.065 |
| rs7751133 | 6 | 167430486 | A | T | NA | 0.085 | 0.017 | 3.73E-07 |  | -0.010 | 0.017 | 0.566 |
| rs77535993 | 8 | 49120463 | T | G | NA | -0.114 | 0.025 | 4.55E-06 |  | -0.039 | 0.025 | 0.109 |
| rs77774500 | 1 | 86852500 | G | A | NA | -0.265 | 0.057 | 3.26E-06 |  | -0.005 | 0.064 | 0.941 |
| rs78064630 | 19 | 10562802 | A | G | NA | 0.176 | 0.031 | 1.08E-08 |  | 0.045 | 0.034 | 0.185 |
| rs78771661 | 8 | 21986850 | T | C | NA | -0.415 | 0.085 | 9.14E-07 |  | -0.070 | 0.123 | 0.569 |
| rs7897792 | 10 | 8108290 | C | G | NA | -0.079 | 0.016 | 1.20E-06 |  | 0.005 | 0.016 | 0.763 |
| rs79051659 | 1 | 151757843 | A | G | NA | 0.161 | 0.026 | 1.30E-09 |  | -0.009 | 0.029 | 0.748 |
| rs7911117 | 10 | 27179596 | G | T | NA | -0.134 | 0.024 | 1.84E-08 |  | 0.033 | 0.023 | 0.151 |
| rs7911680 | 10 | 101293468 | C | A | NA | -0.153 | 0.016 | 6.71E-22 |  | 0.011 | 0.016 | 0.469 |
| rs79541272 | 10 | 133858165 | A | G | NA | 0.113 | 0.024 | 2.07E-06 |  | -0.062 | 0.029 | 0.032 |
| rs798506 | 7 | 2788912 | C | T | NA | -0.121 | 0.018 | 1.47E-11 |  | 0.023 | 0.018 | 0.192 |
| rs8073117 | 17 | 70639396 | A | G | NA | -0.155 | 0.023 | 6.40E-12 |  | -0.007 | 0.021 | 0.734 |
| rs907611 | 11 | 1874072 | A | G | NA | 0.089 | 0.017 | 1.36E-07 |  | 0.016 | 0.016 | 0.333 |
| rs9611131 | 22 | 39662480 | C | T | NA | -0.149 | 0.023 | 5.11E-11 |  | -0.005 | 0.023 | 0.844 |
| rs9960807 | 18 | 12770851 | G | A | NA | 0.112 | 0.023 | 1.44E-06 |  | 0.046 | 0.024 | 0.053 |

Note: SNP, single nucleotide polymorphisms; Chr, chromosome; Pos, position; EA, effect allele; OA, other allele; EAF, effect allele frequency; NA, not available; BETA, effect size; UC, Ulcerative colitis; SE, standard error; *P*, *P* value tests the null hypothesis of no association with exposure.

**Supplementary Table 5. Characteristics of the SNPs used as IVs in the causal association of periodontitis of IBD.**

| SNP | Chr | Pos | EA | OA | EAF | Association with periodontitis | | |  | Association with IBD | | |
| --- | --- | --- | --- | --- | --- | --- | --- | --- | --- | --- | --- | --- |
|  |  |  |  |  |  | BETA | SE | *P* |  | BETA | SE | *P* |
| rs10143801 | 14 | 88321793 | G | A | 0.326 | 0.084 | 0.017 | 8.66E-07 |  | -0.028 | 0.014 | 0.044 |
| rs138868497 | 11 | 86508546 | C | T | 0.010 | -1.639 | 0.332 | 8.20E-07 |  | 0.005 | 0.086 | 0.951 |
| rs151226594 | 11 | 64256137 | G | T | 0.018 | 0.367 | 0.077 | 1.75E-06 |  | 0.006 | 0.052 | 0.913 |
| rs4811024 | 20 | 48909496 | G | C | 0.895 | 0.134 | 0.029 | 4.62E-06 |  | -0.016 | 0.022 | 0.482 |
| rs73155039 | 7 | 136207654 | G | A | 0.011 | -0.832 | 0.176 | 2.22E-06 |  | -0.067 | 0.062 | 0.277 |
| rs76734229 | 18 | 10854213 | A | G | 0.074 | -0.176 | 0.037 | 1.94E-06 |  | 0.043 | 0.024 | 0.073 |
| rs9954920 | 18 | 49752432 | T | C | 0.357 | 0.077 | 0.016 | 2.37E-06 |  | 0.006 | 0.013 | 0.652 |

Note: SNP, single nucleotide polymorphisms; Chr, chromosome; Pos, position; EA, effect allele; OA, other allele; EAF, effect allele frequency; BETA, effect size; SE, standard error; *P*, *P* value tests the null hypothesis of no association with exposure; IBD, inflammatory bowel disease.

**Supplementary Table 6. Characteristics of the SNPs used as IVs in the causal association of periodontitis of CD.**

| SNP | Chr | Pos | EA | OA | EAF | Association with periodontitis | | |  | Association with CD | | |
| --- | --- | --- | --- | --- | --- | --- | --- | --- | --- | --- | --- | --- |
|  |  |  |  |  |  | BETA | SE | *P* |  | BETA | SE | *P* |
| rs10143801 | 14 | 88321793 | G | A | 0.326 | 0.084 | 0.017 | 8.66E-07 |  | -0.055 | 0.018 | 0.002 |
| rs138868497 | 11 | 86508546 | C | T | 0.010 | -1.639 | 0.332 | 8.20E-07 |  | 0.092 | 0.105 | 0.381 |
| rs151226594 | 11 | 64256137 | G | T | 0.018 | 0.367 | 0.077 | 1.75E-06 |  | -0.001 | 0.067 | 0.985 |
| rs4811024 | 20 | 48909496 | G | C | 0.895 | 0.134 | 0.029 | 4.62E-06 |  | -0.058 | 0.029 | 0.044 |
| rs73155039 | 7 | 136207654 | G | A | 0.011 | -0.832 | 0.176 | 2.22E-06 |  | -0.055 | 0.079 | 0.482 |
| rs76734229 | 18 | 10854213 | A | G | 0.074 | -0.176 | 0.037 | 1.94E-06 |  | 0.033 | 0.031 | 0.282 |
| rs9954920 | 18 | 49752432 | T | C | 0.357 | 0.077 | 0.016 | 2.37E-06 |  | 0.002 | 0.017 | 0.897 |

Note: SNP, single nucleotide polymorphisms; Chr, chromosome; Pos, position; EA, effect allele; OA, other allele; EAF, effect allele frequency; BETA, effect size; SE, standard error; *P*, *P* value tests the null hypothesis of no association with exposure; CD, Crohn’s disease.

**Supplementary Table 7. Characteristics of the SNPs used as IVs in the causal association of periodontitis of UC.**

| SNP | Chr | Pos | EA | OA | EAF | Association with periodontitis | | |  | Association with UC | | |
| --- | --- | --- | --- | --- | --- | --- | --- | --- | --- | --- | --- | --- |
|  |  |  |  |  |  | BETA | SE | *P* |  | BETA | SE | *P* |
| rs10143801 | 14 | 88321793 | G | A | 0.326 | 0.084 | 0.017 | 8.66E-07 |  | -0.009 | 0.018 | 0.616 |
| rs138868497 | 11 | 86508546 | C | T | 0.010 | -1.639 | 0.332 | 8.20E-07 |  | -0.127 | 0.116 | 0.273 |
| rs151226594 | 11 | 64256137 | G | T | 0.018 | 0.367 | 0.077 | 1.75E-06 |  | 0.036 | 0.066 | 0.579 |
| rs4811024 | 20 | 48909496 | G | C | 0.895 | 0.134 | 0.029 | 4.62E-06 |  | 0.019 | 0.028 | 0.515 |
| rs73155039 | 7 | 136207654 | G | A | 0.011 | -0.832 | 0.176 | 2.22E-06 |  | -0.072 | 0.079 | 0.362 |
| rs76734229 | 18 | 10854213 | A | G | 0.074 | -0.176 | 0.037 | 1.94E-06 |  | 0.066 | 0.030 | 0.030 |
| rs9954920 | 18 | 49752432 | T | C | 0.357 | 0.077 | 0.016 | 2.37E-06 |  | 0.011 | 0.017 | 0.513 |

Note: SNP, single nucleotide polymorphisms; Chr, chromosome; Pos, position; EA, effect allele; OA, other allele; EAF, effect allele frequency; BETA, effect size; SE, standard error; *P*, *P* value tests the null hypothesis of no association with exposure; UC, Ulcerative colitis.

**Supplementary Table 8. GWAS summary statistics comparation of two MR.**

| GWAS | | Sample size | Case | Control | Cases/Sample size | Population source |
| --- | --- | --- | --- | --- | --- | --- |
| IBD | Our study | 59957 | 25042 | 34915 | 0.42 | Canada, UK, and 12 more countries |
|  | Wang et al | 34652 | 12882 | 21770 | 0.37 | Canada, UK, and 12 more countries |
| UC | Our study | 45975 | 12366 | 33609 | 0.27 | Canada, UK, and 12 more countries |
|  | Wang et al | 20883 | 5956 | 14927 | 0.29 | Canada, UK, and 12 more countries |
| CD | Our study | 40266 | 12194 | 28072 | 0.3 | Canada, UK, and 12 more countries |
|  | Wang et al | 27432 | 6968 | 20464 | 0.25 | Canada, UK, and 12 more countries |
| Periodontitis | Our study | 45563 | 17353 | 28210 | 0.38 | UK, U.S., Germany and Sweden |
|  | Wang et al | 198441 | 3046 | 195395 | 0.02 | Finland |

Note: IBD, inflammatory bowel disease; UC, ulcerative colitis; CD, Crohn's disease; MR, Mendelian randomization; The population of the GWAS of IBD, UC and CD includes Canada Denmark, UK, Italy, Lithuania, Baltic, New Zealand, Norway, Spain, Germany, Australia, Sweden, USA, Belgium, Slovenia, Netherlands and UK
